# Energy gamification: the development of a user interface tool to upgrade social experience and energy literacy

**DOI:** 10.12688/openreseurope.15158.1

**Published:** 2022-11-28

**Authors:** João Cravinho, Ricardo Lucas, Miguel Brito, Daniel P. Albuquerque, Uways Mithoowani, Nuno M. Mateus

**Affiliations:** 1 EDP NEW R&D, Lisbon, 2685 – 039, Portugal; 2 Sapienza Università di Roma, Piazzale Aldo Moro, Roma, 00185, Italy

**Keywords:** serious games; gamification; energy; energy efficiency; energy consumption; energy conservation; user engagement.

## Abstract

Gamification consists in the application of typical elements of game-playing environments to other areas of activity. In different fields such as medicine, education, or business, gamification has been explored as an efficient vehicle to foster real-life predetermined targets or improve a real-life action effectiveness. Amidst the current energy transition, gamification is one of the available strategies to make the energy transition exciting to the end-user, proposed in recent times as a means of bridging information gaps, increasing learning, and motivating behaviour-change. The ultimate goal of using gamified solutions is not to influence the user to save energy with the goal of an extrinsic reward, however intangible, but to save energy because they have come to see it as intrinsically satisfying and meaningful. Leveraging in the increasing digitalization of the energy sector, gamified solutions can provide a useful user-engagement platform while fostering energy-consumption behavioural-change. Hence, in this context, the Smart2B H2020 project aims to present and analyse how can these gamified solutions create an excellent user-engagement experience while encouraging and fostering energy literacy and behaviour-change. The developed gamified module will comprise a user interface (UI) tool where a healthy competition between users will take shape – driven mainly by the user’s energy consumption behavioural change – and the monthly and overall leader boards will translate the energy savings achieved by the users into an in-game virtual point-based system. Other gamified elements and mechanisms such as missions, interactive tasks or challenges, instant feedback or achievements, and badges will further progressively guide the user through their energy consumption patterns and how they can be improved. A social engineering and educational perspective, brought possible within the context of the pilot sites in Smart2B’s project, will focus on the maximization of user interaction and engagement and how can these gamified solutions motivate real behaviour-change.

## Plain language summary

Gamification, which consists in the application of elements which are part of game environments (such as points, leader boards, levels or stages, missions, and achievements) to real-life situations, bringing forth the motivational potential of game environments and enhancing and addressing real-life objectives. In the context of the Smart2B project, gamification will take shape in a virtual game environment – the Smart2B platform – where end-users will be engaged and incited to optimize their energy consumption patterns. By fostering a healthy competition based on a in-game point system, users will face different tasks and interactive challenges in order to foster energy conservation, increase energy literacy and ultimately to enhance user-engagement. Combining the energy consumption behaviour change while measuring the user engagement with the gamification mechanisms one can draw insights on the effectiveness of gamification.

## 1. Introduction

Human brains are wired in such a way that people enjoy engaging with challenges and platforms, reaping the positive feedback, rewards, and the social-bonding perspective that games provide. They are one of the most widespread strategies to which human beings’ resort to either interact, communicate, or simply have fun. With the advent of digital technology, games have become even more accessible to people – recently, video games have become increasingly popular among all ages and gender groups, often regarded as the “central entertainment media of the future” ( McGonigal, 2011). In order to differentiate the large number of games and video games, and to make them more compelling, current games rely heavily on user engagement mechanisms, such as points, badges, a compelling narrative, or virtual in-game currency. With the increasing capacity to generate and process data the paradigm of purely-entertainment driven games has shifted to the increase of ‘serious games’ – a non-entertainment focused game, where the primary purpose is to foster some kind of predetermined action or activity (e.g., such as improving the learning experience), instead of hedonic games ( Brackenbury & Kopf, 2022). There is no doubt about the inherent motivational potential that video games, and games in general, possess. This potential has been extensively covered in literature and explored in serious gaming ( Khan *et al.*, 2020). Expanding on the concept of resourcing to gamified elements to improve an activity’s efficiency, gamification has surfaced to bring the motivational power of video games to real-life and real-world applications. Gamification imports elements, mechanics, design, and principles of game-theory and game environments into other areas of activity, usually real-world contexts, transforming everyday real-life activities into game-like experiences ( Beck *et al.*, 2019). Since its wide adoption, from 2010 onwards, gamification has extended to pretty much all areas of human activity – from work ( Ferreira-Oliveira *et al.*, 2017), to medicine ( Sardi *et al*., 2017), education ( Nevin *et al*., 2014), or even within the energy sector, gamified solutions are increasingly being explored as an efficient instrument to engage with users and achieve real-life targets. Gamification is indeed deemed as providing positive effects, despite being “greatly dependent on the context in which the gamification if being implemented, as well as on the users using it” ( Hamari *et al*., 2014). Studies ( Beck *et al.*, 2017) also indicate that providing information through gamified solutions may increase its impact in comparison to common communication channels. In the context of the current energy transition and leveraging in the increasing digitalization of the energy sector, gamified solutions can provide a useful user-engagement platform while fostering energy-consumption behavioural-change. The European building stock is currently responsible for almost 40% of final energy consumption and 36% of the final CO _2_ emissions globally ( European Comission (EC), 2020). Adding to the fact that people spend a large amount of their time inside buildings and that around 75% of the current EU-27 building stock is “energy inefficient” ( Lewis *et al*., 2021), it serves as an effective vehicle of change for the energy transition targets ( European Comission (EC), Directorate-General for Climate Action, 2019). In this context, the Smart2B H2020 project ( Smart2B H2020 Project, 2022), which aims to upgrade the smartness levels of existing buildings through coordinated cloud-based (i.e., Smart2B platform) control of legacy equipment and smart appliances while offering new energy and non-energy services (e.g., increased energy efficiency, improved indoor comfort to the occupants and flexibility) to various stakeholders, will be able to provide a testbed to answer the broad research question of ‘how can the energy transition in buildings be leveraged by gamified solutions’.

The aim of this study is to present and analyse how can gamified solutions create an excellent user-engagement experience, while encouraging and fostering energy literacy and behaviour-change, in the project’s context. The developed gamified solution will comprise a user interface gamified module where a healthy competition between users will take shape – driven mainly by the user’s energy consumption behaviour and behaviour-change – and the monthly and overall leader boards will translate the energy savings achieved by each user into an in-game virtual point-based system. Other gamified elements and mechanisms such as alternate missions, interactive tasks or challenges, instant feedback, badges, and the interaction with Smart2B’s innovations will further progressively guide the user through its energy consumption patterns and how they can be improved. A social engineering and educational perspective, brought possible within the context of the pilot sites in Smart2B, will focus on the maximization of user interaction and engagement and how can these gamified solutions motivate real behaviour-change.

Section 2 details a literature review of gamified solutions applied in the energy sector’s context, in 2.1, which paves to a description of the developed gamified concept and component, in Section 3. In Section 3.1 the different and relevant game design elements considered within the context of the developed gamification component are detailed. Section 4 includes the discussion and final remarks.

## 2. Gamification in the energy sector

Through strategic plans such as the European Green Deal ( European Comission, 2019), the “Renovation Wave” ( European Comission, 2020), and the recent REPowerEU ( European Comission, 2022) the European Union is increasingly committed to developing a sustainable, secure, competitive, and decarbonized energy sector by 2050. To achieve such goals, special focus should be given to the building sector which accounts for almost 40% of final energy consumption and 36% of the final CO _2_ emissions ( European Comission (EC), 2020) and is among the largest end-use consumer sectors ( D'Agostino *et al.*, 2017). The building sector’s energy consumption reduction may be achieved by different means such as the adoption of building energy efficiency standards, promoting building renovation or resourcing to digital and ICT solutions for building automation and response, among others ( Casals *et al.,* 2020). Findings ( Zhao *et al.*, 2017) show that along with the ever increasingly capacitating technological advances in buildings systems, inciting the end-user’s engagement and behavioural change is key. Hence, gamification can be explored as an effective mechanism to further engage end-users and ultimately foster behavioural change ( Fijnheer & van Oostendorp, 2016). Additionally, gamified energy-related solutions/applications can bring “significant value streams to residential customers while driving positive and measurable business outcomes for energy suppliers and society as a whole” ( AlSkaif *et al.*, 2018).

The next Section 2.1 reports previous studies which focused on gamified solutions tackling energy consumption and how can they maximize user engagement. In Section 3 the Smar2B’s gamified solution is described: in Section 3.1 a description of the use case at hands, followed by a detailed description of the gamification module concept and components.

### 2.1. Gamification in the energy sector

By bringing the motivation enhancement aspect of game environments to the real-life demand-side energy system environment, it is possible to further address the energy transition targets [12] within the ineffective ( Lewis *et al.*, 2021) and energy-intensive ( European Comission, 2019) European building stock, while motivating real-life behavior change in the building’s consumption patterns and fostering energy literacy among end-users. Hence, successful gamification within the energy sector must act in two distinct fronts: incite the users’ short-term engagement, by fostering some real-life benefits which act as incentives (extrinsic motivation), without neglecting the much needed long-term engagement, by building the intrinsic motivation, unlocking the possibility to motivate real-life energy consumption behavior change ( Wee & Choong, 2019). The diverse panoply of game design, principles, elements, and mechanisms (see Section 1) that are brought to the real-life environments constitute the building blocks of gamified solutions, as the game design elements ( Sailer *et al.*, 2017). They represent different mechanisms through which different motivational outcomes are triggered. For the engagement to succeed, these motivational outcomes must be compliant with the users’ needs and target them: the users’ behavioral constructs and psychological needs ( Frederiks *et al.*, 2016) towards energy consumption must be addressed *via* the game design elements. Despite the many gamified applications found to target energy consumption in buildings, energy consumption, and sustainability ( Johnson *et al.*, 2017) there’s still questions raised about its real and actual effectiveness ( Grossberg *et al.*, 2015). The game design elements which are most found in the application of gamification within the energy sector are: points, badges, missions, rankings and information provision, all which target different motivational outcomes ( AlSkaif *et al.*, 2018). According to ( Grossberg *et al.*, 2015), gamification can unlock 10% of energy consumption savings to end-users.

## 3. Smart2b gamification concept

**Figure 1.**
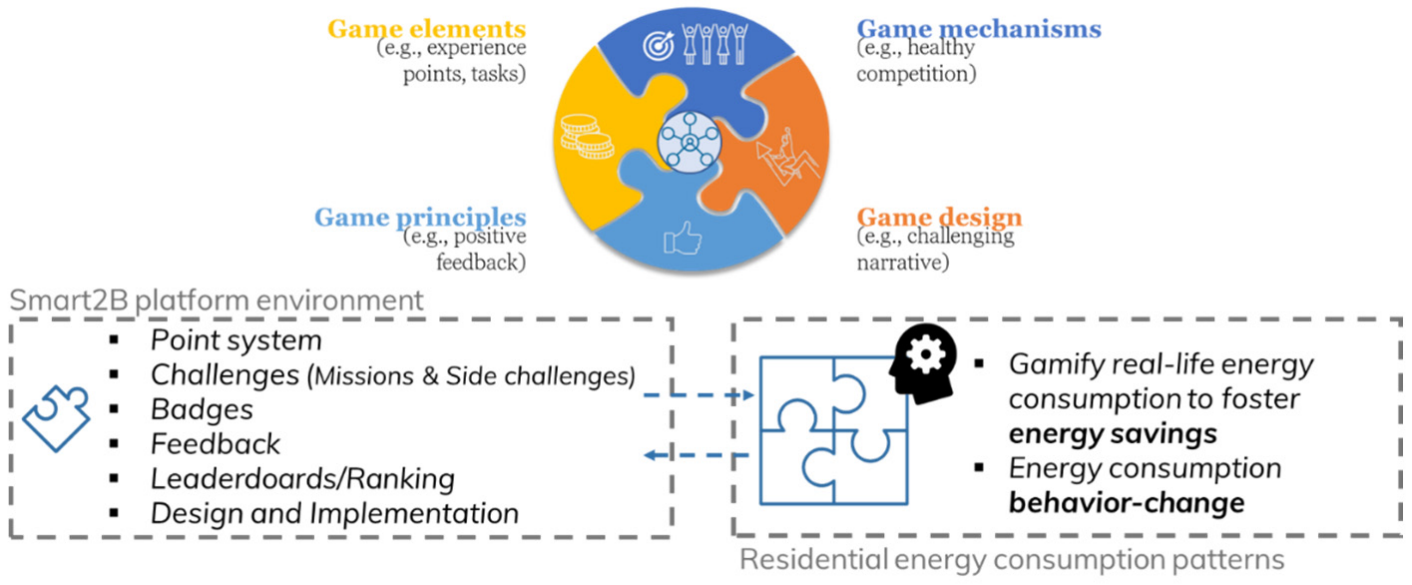
User centred approach for the conceptualization of the game-design elements (above), which compose the Smart2B’s modular gamified solution (schematic representation below).

Attending on the work developed within the consortium concerning the specification and requirements for the Smart2B concept, and specifically the stakeholder framework ( Croé, 2022) it was possible to narrow down the scope of the gamified solution’s concept. Tailoring the gamified module concept to the specifications of the demonstration activities, the gamified solution will focus on promoting a cooperative competition environment focused on achieving energy savings and energy consumption behaviour-change among the platform’s users. Missions, smaller interactive challenges, instant feedback, and badges will translate real-life actions into an in-game experience point (XP) system, through which user’s will be ranked according to their performance. Different competitions will be fostered, aiming to tap into different motivational triggers: a competition between individual users and a competition where different occupants of the same residential building will compete against other residential ‘clusters’ (i.e., other residential buildings which can have multiple occupants). Through an increasingly challenging and progressive game narrative, and the interaction with the Smart2B innovations, the users will be progressively guided through their energy consumption patterns and how they can be improved. An educational layer to the game design elements, will provide an additional interaction and engagement platform while fostering real and lasting behaviour-change. In Figure 1 a schematic representation of the iterative and incremental user-centred approach for the design of gamified solution: all game design elements considered are tailored to the Smart2B users. In the same Figure 1, the overall gamification component concept is schematized, below.

Together with the work developed within the consortium ( Croé, 2022) and leveraging the synergies between consortium partners, the modular component was conceptualized and validated through a set of functional prototypes, developed in Figma ^†^, which were then tested with potential Smart2B users. These prototypes serve the purpose of illustrating the game design elements while also assisting in generating a list of functional requirements for the actual platform development. The gamification module will be coupled and seamlessly integrated in the User-Interface (UI), being developed within the consortium for the purpose of establishing the communication channel between the Smart2B platform and its end-users, involved in the demonstration activities. The UI description and development process are documented in ( Santana & Fonseca, 2022). Hence, along with the Smart2B’s gamified solution’s concept, the game design elements which compose the gamified solution are to be developed in the existing cross-platform framework (Meteor ^‡^) – i.e., both the back (Node.js ^§^) and front-end (React ^**^) developments required to operationalize the gamified solution.

### 3.1. Use case description

The gamified environment and the gamification module, operating within the Smart2B platform, is one of the project’s use cases, focused on bridging the information gaps between users and the Smart2B platform, services and UI. Conceived with a clear user-centred approach, as schematized in Figure 1, the UI will be tailored to each users’ needs – the level of interaction and automation of the UI is tailored to what “each user demands from the system” ( Albuquerque *et al.*, 2022).

Within the project different actors will communicate via the dashboards and UI with the Smart2B platform. Each actor – building managers, occupants, grid operators and (groups of) citizens – will have different profile types, and consequently different functionality levels, within the virtual environment, ensuring that “all actors will only see relevant information for them so only the needed functionalities and data will be presented to all of them” ( Albuquerque *et al.*, 2022).

**Figure 2.**
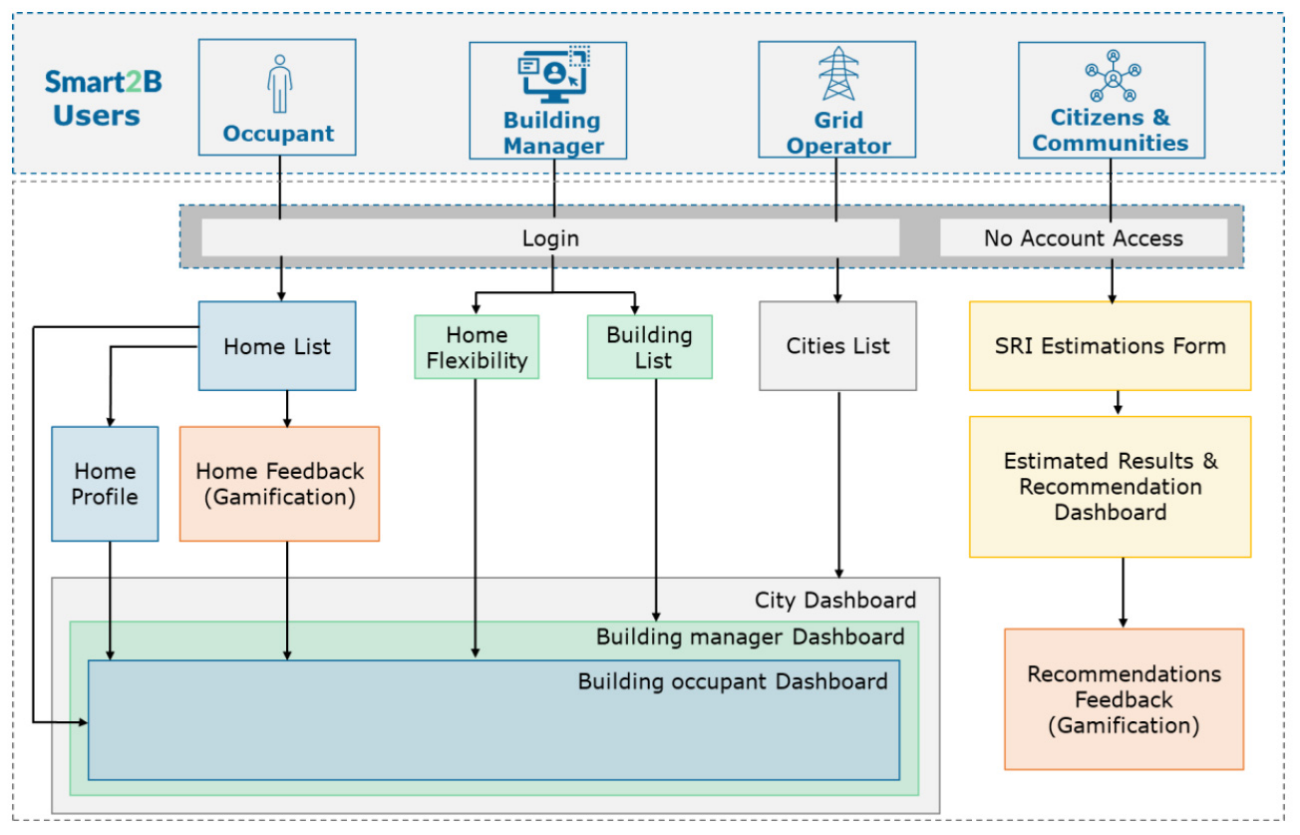
Use case diagram. Removed from ( Albuquerque *et al.*, 2022).

**Figure 3.**
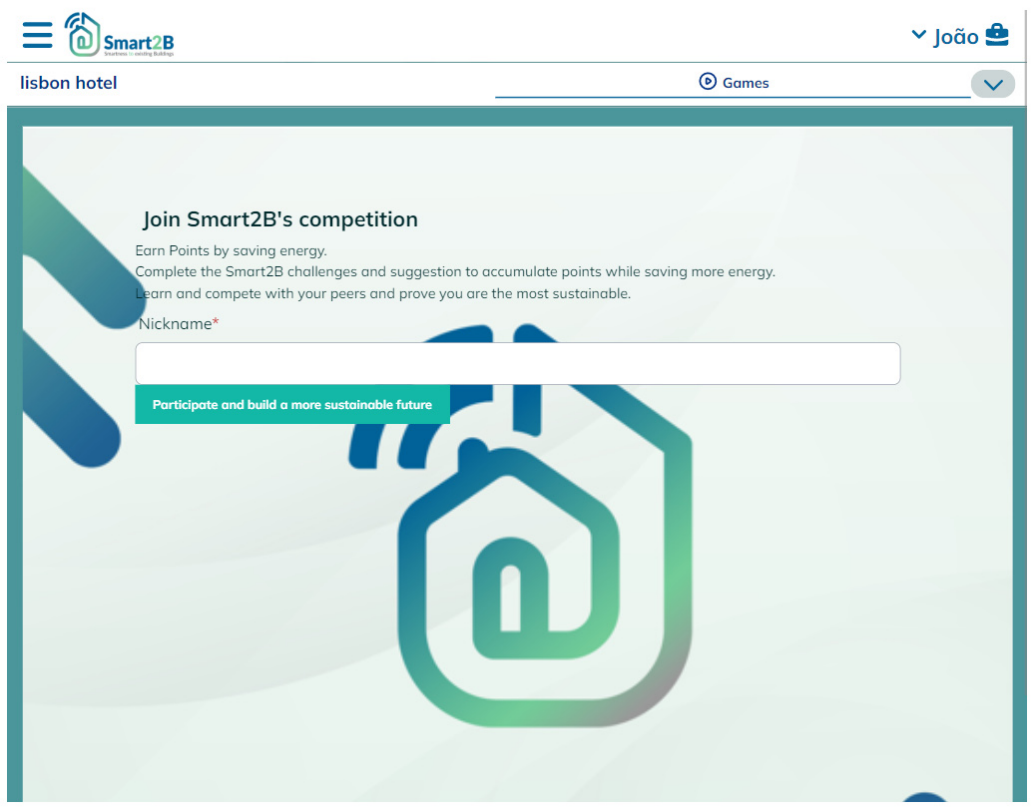
Meteor prototype of the introductory (Hero) page displayed to first-time users of the gamification module.

Building managers will be able to access a list of all the buildings they manage, while also having the possibility to add new ones – when “adding a new building (…) the user will be asked about some data regarding building identification and to enter the maximum and minimum values for each of the three dimensions that the occupants can adjust” ( Albuquerque *et al.*, 2022). The occupants will be able to monitor, in real-time, the energy flows and the Smart Readiness Indicator (SRI) level of their house (taking into consideration the possibility of each occupant user having more than one residency), while also being able to control three dimensions: comfort, energy savings and environmental impact – since “manipulating one of the dimensions will affect the others, users must accept the trade-off” ( Albuquerque *et al.*, 2022). Grid operators will be able to access and monitor the flexibility services “proposed to consumers are being used/accepted by them” ( Albuquerque *et al.*, 2022), with an on-demand granularity possibility (i.e., from cities to apartments). Citizens or groups of citizens will be able engage and access the Smart2B platform to estimate their homes/buildings SRI level. A descriptive diagram of the use case can be found in Figure 2, below. A more detailed description ( Figure 3) of the use case can be found in ( Albuquerque *et al.*, 2022).

The different game design elements within the virtual environment of the Smart2B platform, described in the upcoming section, with which the users will be able to interact will be monitored alongside the energy consumption patterns. The interaction level of the users with the different gamified mechanisms can then be compared with the monitoring of the end users’ energy consumption patterns to draw insights on the effectiveness of the different game design elements.

To collect user feedback regarding the gamification component’s functionalities and its overall feel and look, local workshops will take place with groups of users, belonging to the different Smart2B group of actors. The users will be provided a script, guiding the users through the app’s virtual environment highlighting some of the key design elements (see Section 3.2), and a set of linear numeric scale and open questions to evaluate their experience (see Supplementary Material).

### 3.2. Game design elements

When users navigate through the UI to the gamification module for the first time they’ll be presented with an initial ‘Hero Page’ ( Figure 3). This page will contain an introduction to the gamification module: the clear guidelines, rules of play, and goals of the gamified mechanisms and elements which the users will face, as well as the benefits (individual *vs* collective and real-life *vs* virtual) of participating in the designed gamified solution. The highlighted information will speak to the core of the gamification concepts explored in 2.1 (e.g., the users psychological needs) while also emphasizing the different and achievable benefits, which can act as incentives to users ( Beck *et al.*, 2019) – whether it’s from an individual perspective or from a community point-of-view the economic, environmental, or social incentives and benefits can be tapped and enhanced by gamified solutions ( AlSkaif *et al.*, 2018).

**Figure 4.**
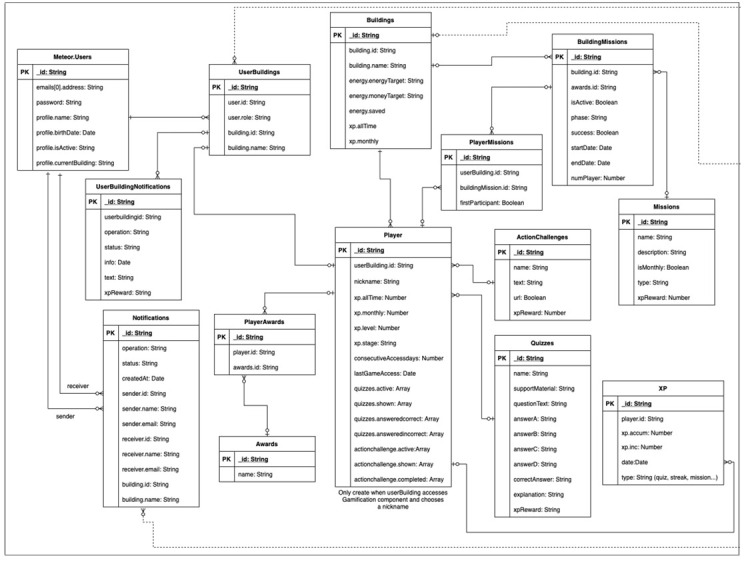
Back-end data model for the Smart2B user interface (UI) and gamification components.

Upon clicking the displayed button (in Figure 3, represented by the green ‘Participate’ button), a game profile will be created for the user – a mongoDB data collection, automatically generated for each new player (i.e., an association between a user and a building). The player profile will figure every back-end piece of information, data, or variables associated with the user, and associated building, which needs to be transferred among game design elements (e.g., users’ unique identification, the building’s unique identification, the amount of points the user accumulated so far, etc), ensuring consistency in the data model throughout the different game design elements implemented. In Figure 4, the data model structure for the gamification module and UI is presented. The data model, alike relational databases, is composed of tables and relationships between them through primary keys, PK, avoiding data overlapping and duplication – each table groups different variables of the same nature.

**Figure 5.**
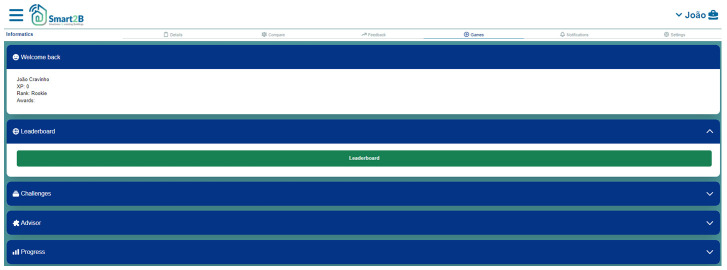
Smart2B's gamification module homepage prototype, developed in Meteor.

Consequently, the user will be forwarded to the gamification module homepage as shown in Figure 5. In the next sections the main game design elements included in the Smart2B’s gamification module are detailed.

**Figure 6.**
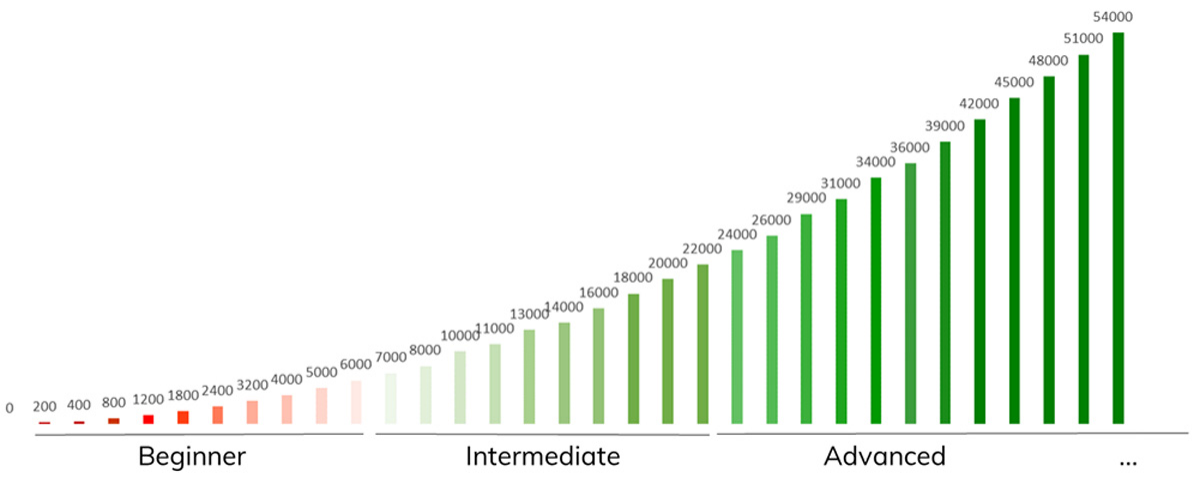
Schematic representation of the Smart2B gamified narrative: each experience stage (Beginner, Intermediate and Advances, below) is composed of 10 intermediate levels. Each bar represents one level, displaying above the accumulated XP points required to reach it.

***3.2.1. In-game point system*.** The designed in-game environment point system is the main building block of the gamification concept. Points are to be rewarded to a user as an in-game consequence of successfully completing a task, request, or to reward engagement. The experience point (XP) system is composed by two main components, levels, and stages, creating an incremental and progressive environment and narrative. The schematic representation of the designed experience point system is shown in Figure 6.

There are three stages which represent big milestones for the user:

▪ Beginner: the user begins its journey on the first stage, the ‘beginner’ stage, where they must learn how to work with the application basic features. Here it is recommended to provide initial information to the platform, as well as provide feedback regarding equipment usage, preference settings, among others. These actions ensure that the player is comfortable with the whole gamification module and platform.
▪ Intermediate: after gaining 6000 XP, which should translate into around six months of consistent usage, the user ‘levels up’ to the second stage, ‘intermediate’. Here they should be a knowledgeable user of the platform and are encouraged to improve their energy saving with weekly or daily goals. At the same time, an increase in knowledge around energy-related topics is facilitated with the help of quizzes, topical questions and informative videos (see the educational challenges, described under Section 3.2).
▪ Advanced/Ambassador: at 24000 XP, approximately 18 months of app usage, the user arrives at the third and final stage, where they are a proficient energy saver. At this stage, the user is still encouraged to improve the energy efficiency of the household while deep diving more seriously into related energy topics. It is expected, that at this stage, the user no longer needs the gamification component of the application to ensure that they maintain their behaviour, therefore this component has had the desired effect of instilling the intrinsic behaviour which promotes energy savings, (practical and non-practical) sustainability-driven actions, and knowledge.

The other experience point system gamified mechanism are the levels. The levels give the user an incremental sense of growth and improvement in the platform. Operating in a smaller scale than the stages, the levels are more easily attainable and achieved, keeping the user engaged. Each stage is divided by 10 levels with incremental gaps of XP to ensure that the user feels a continuous experience throughout the game narrative, with the exception of the third stage (Advanced) which has no limit to the number of levels, ensuring that the game does not come to an end and that the users will have the continuous experience until the end of the project’s demonstration actions.

Two different entities can ‘earn’ experience points: the user and the building, in which the users live or work. Buildings XP can only be obtained through challenges directly related to energy savings, while the user is also encouraged to strengthen their knowledge and give feedback to the platform by earning experience points from all gamified challenges (see Section 3.2).

**Figure 7.**
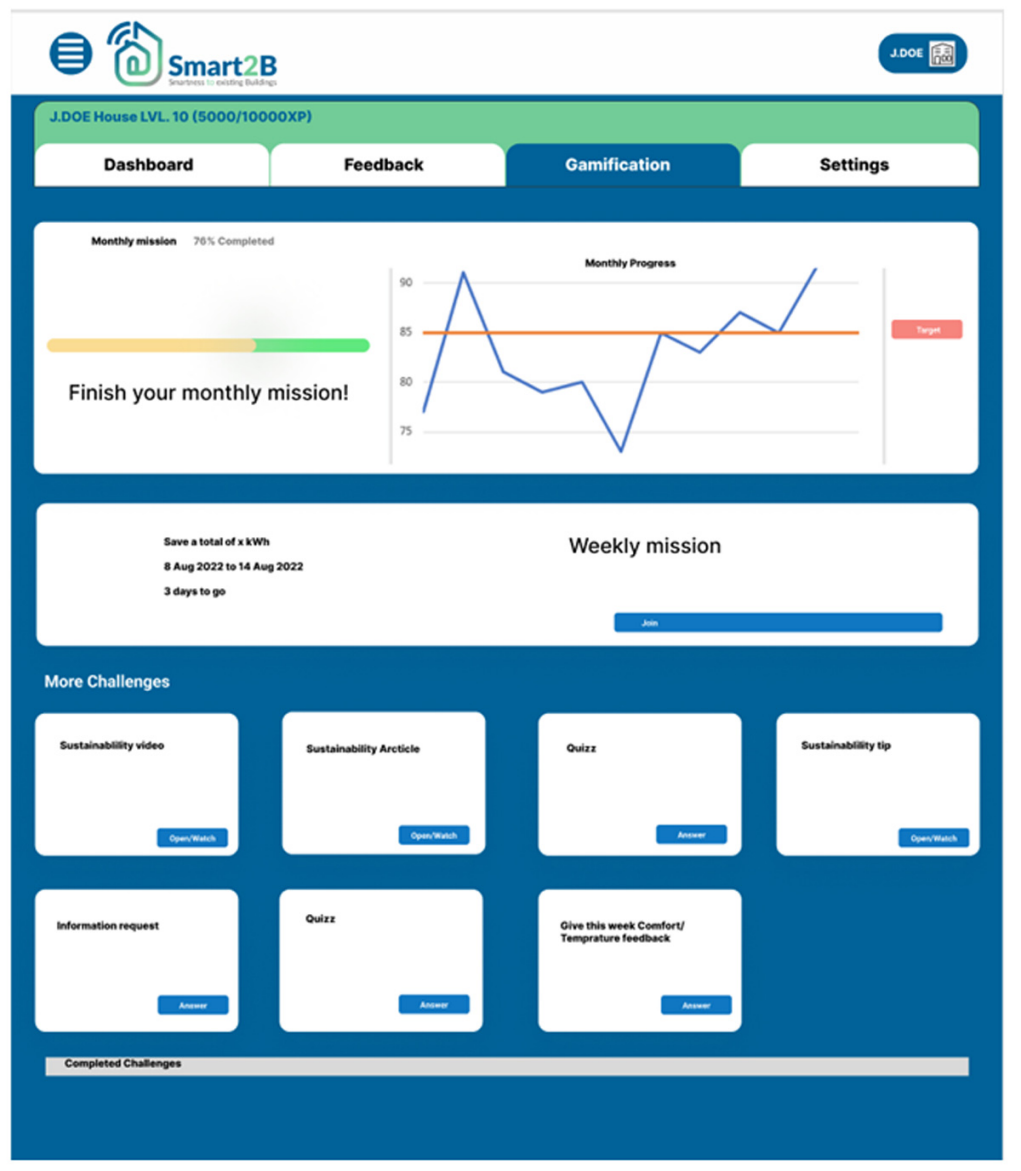
Figma prototype of challenges section of the Smart2B gamification module.

***3.2.2. Gamified challenges*.** Anchored and leveraged by the in-game experience point (XP) system described above, users will be faced with interactive challenges which tap into different motivational triggers or incentives. Five different gamified challenges are considered: missions, information requests, quizzes, videos and articles, each rendering different points to the user. Figure 7, displays a prototype of the challenges section of the gamification module.

The user will be able to see the various gamified activities, challenges, and missions which will further address and contribute to the short and long-term engagement while addressing different motivational triggers. Despite the prototype, displayed in Figure 7, showing all types of challenges, only four at a time will be displayed to the user. It is possible to categorize the challenges by their main theme:

○ Missions: Two missions are to be considered. The main monthly mission, worth 200 points, is always related to lowering energy consumption compared with the previous month’s consumption. This mission increases in difficulty to keep up with the user level of expertise and knowledge. The main objective of this monthly task is to keep the user focused on the topic of lowering energy consumption as the main goal of the gamification application, without hindering the users’ comfort level. The weekly mission, similar to the previous, focuses solely on the energy consumption of the user. Through various prompts such as minimizing consumption for a day below a certain value, minimizing overall weekly consumption or establishing a comparison between users’ energy consumption metrics, this weekly challenge makes sure that the user can feel the benefits of saving energy not only at the end of the month, but on a weekly basis. This challenge will be worth 75 XP, with a bonus of 10 XP for the first user to join the mission. The main, monthly, mission will run through each month – since the first day of the month until the day before the end of the month –, while the weekly mission will run from every Monday to each Saturday morning, giving time to every user to be aware of the next week’s mission content, speaking to the challenges’ discrete timeline guideline, as described in the previous Section 2.1.

The remaining challenges will be composed by three rotating weekly challenges which will more heavily depend on the user level, in order to guide them through the platform at a suitable pace, not letting the user feel overwhelmed at the beginning of the experience or eventually leading to boredom with a lack of tasks to perform. These side missions will be worth from around 20 to 40 XP, depending on difficulty, to which certain bonus might be added. We can distinguish two types of challenges:

○ User focused challenges: Tasks like navigating and displaying specific information within the Smart2B platform, especially during the beginning of the demonstration activities, such as energy consumption, generation, or flexibility, belong under this category. The user is encouraged to continue to provide feedback throughout the project – relative room temperature and humidity are some of the data required. These tasks will reward the user with 25 XP.

○ Instructional challenges: The last type of missions will be mostly informative and educational, this includes both quizzes and single questions, as well as informative videos for the users to learn more about certain topics. These tasks are more time consuming and involve a more active participation by the user, therefore the successful accomplishment will be reward 40 XP.

Additionally, the user can also be attributed a bonus of 50 experience points by completing all four weekly challenges (with the exception of the main monthly mission), making sure that they are encouraged to keep completing tasks after the monthly mission or even if the tasks have a higher degree of complexity.

***3.2.3. Leader boards*.** The goal of this section is to let the user know how they compare with the overall population that is also playing the game and giving them a goal to strive for, bringing the motivational triggers to real-life and inciting users to improve their experience points by completing challenges and to improve their household by saving more energy, furthering user-engagement. The leader boards present player name, current stage, current level and XP or the building’s normalized energy consumption (kWh/m ^2^). The goal is for users to strive to be top of both the overall ranking, as well as the monthly one, either by themselves or with their building (energy-related and progress-related), to accrue more XP.

**Figure 8.**
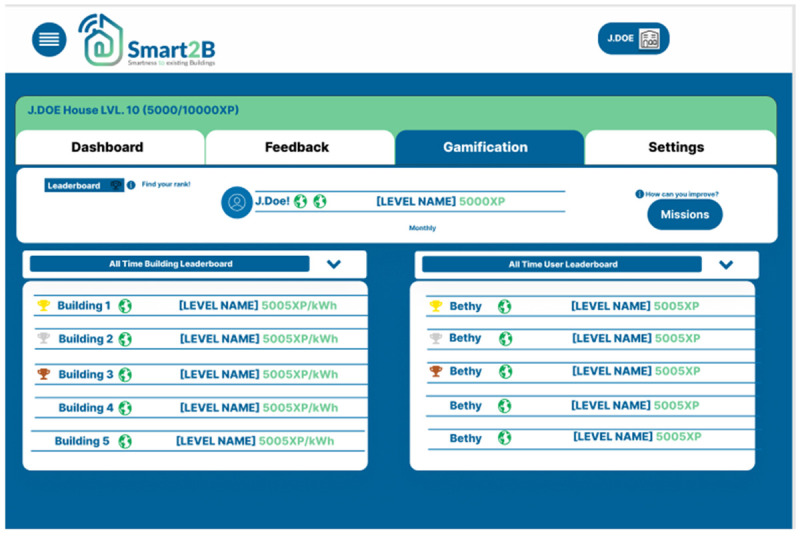
Figma prototype of leader boards section of the Smart2B gamification module.

This section is divided in two main leader boards, one which is focused on the user and one focused on the buildings. In the building leader board, only buildings are compared with each other, either by XP gained from the monthly and weekly mission only, or by their normalized energy consumption, in the form of kWh/m ^2^. The building competition serves the purpose of creating a common goal for all occupants of the same building and to compete with other buildings. The user leader board is used to compare the XP accumulated by the users, with an option to compare yourself to all users participating in the competition or to the other Smart2B platform users which are associated to the same building, inciting a friendly competition between users in the same conditions. Both classifications have the option to see the all-time comparison between members or to just compare a single month. This option allows the user to see the progress made in each month. Figure 8 shows the prototype for the web section, illustrating the different functionalities of the leader boards and the different layers of competitions.

***3.2.4. Engagement and rewards*.** The user is incentivized and rewarded for continuously interacting with the Smart2B gamification module – by completing tasks, accessing every day or by the continuous improvement made throughout the gamified narrative. Besides the bonus rewards described in 3.2, in which the user can accumulate bonus points if all weekly challenges are successfully completed and a bonus XP for the first user who subscribes to the weekly mission, different engagement and rewarding mechanisms are contained within the Smart2B gamified solution:

Firstly, a ‘login’ streak counter informs and rewards users for continuously accessing the gamified module. For each consecutive day the user accesses the platform, the user accumulates additional five XP. By accessing the module in consecutive days, the user will be rewarded with a bonus of XP points, proportional to the number of consecutive days they have accessed the gamified module. By accessing two days in a row a user will be awarded five XP points, while at the seventh consecutive day the bonus increases to 50.

Secondly, an end of month bonus rewards the user for all the improvement made during that timeframe. Throughout the month, the user’s saved energy and process through the leader boards is calculated and, along with the earned experience points, earn the user a monthly bonus. This bonus depends on leader board position in terms saved energy, in kWh, and on the experience (XP) points achieved during that month: for every 100 XP won during the month, the user gains an extra five experience points.

Finally, badges will not render any XP to the users, and they are awarded to signal certain achievements or milestones. Finishing missions, saving a certain amount of energy (Wh or kWh), successfully completing challenges, being the first in the leader board, among others, are all ways of earning badges that represent the user’s achievements and improvements throughout the gamification component’s narrative.

**Figure 9.**
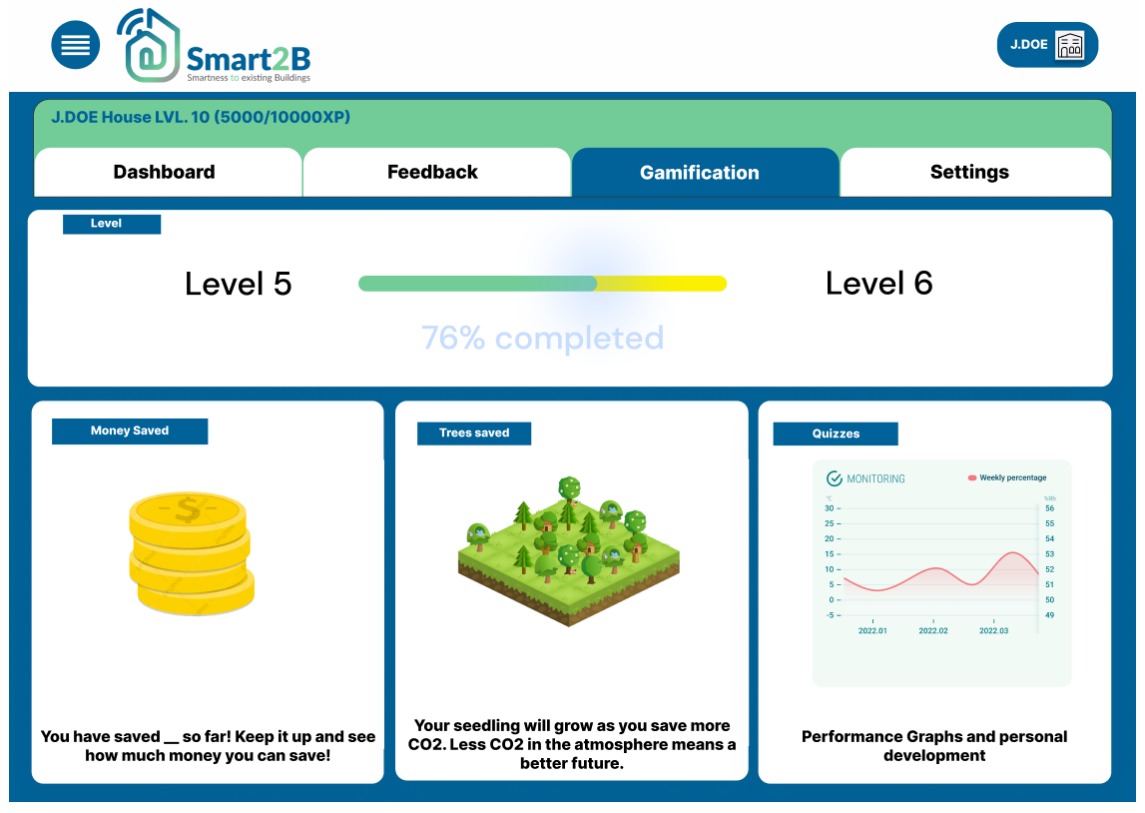
Figma prototype of progress section of the Smart2B gamification module.

***3.2.5. Progress*.** The information feedback loop, crucial to keeping users engaged, will take shape within the progress section. This section gives easily accessible information to the user about its improvement and achievements. Here the user can see information about the current level and points accumulated, the amount of energy and money saved and the progress throughout the challenges which the user is faced with. This section of the component focuses ( Figure 9) on providing the intrinsic reward and motivation to the user, through three different incentives, each with the focus to show users the benefits the user has been able to achieve during its participation in Smart2B’s gamified competition: monetary savings, emissions savings and personal development:

▪ Personal development: the user can see how much XP they have earned so far, as well as how much XP is left for the next level and stage. The information feedback will relate to the overall progress of the user since the beginning of usage of the gamification component in terms of accumulated XP. This will reinforce positive feedback to the user, inciting a continuous use of the application.

▪ Monetary incentives: a conversion from energy saved into money saved is considered. Here, depending on the energy tariffs the user benefits from, it can be seen the monetary benefits of using smart appliances and managing energy consumption. Monetary benefits can be a big persuasive for users to implement new tasks and improving the smartness of the building.

▪ Emissions savings: the energy saved is converted into saved trees. A CO _2_ to trees convertor will be used to let the user know how much they are helping the atmosphere and the whole planet. Similarly, to the monetary benefits, the environmentally friendly mentality is a good one to try to implement on our userbase, which might in the future lead them to adapt more environmentally friendly practices.

**Figure 10.**
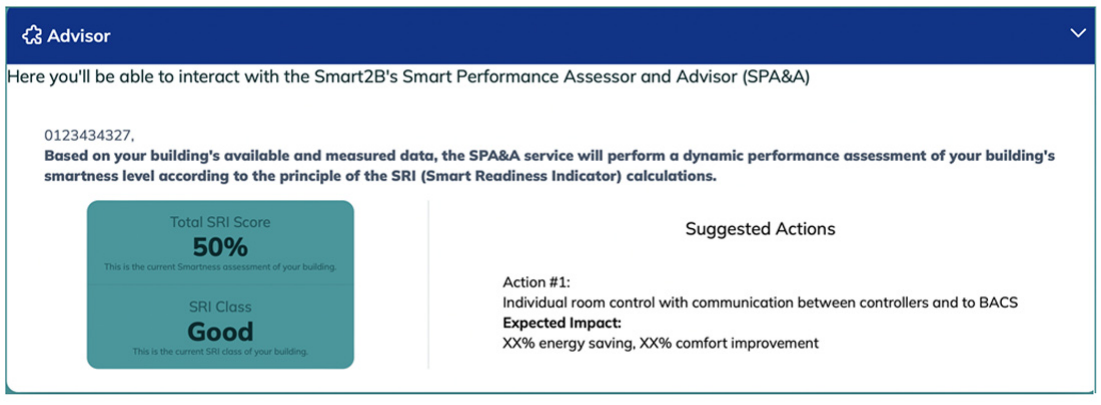
Meteor prototype of the Smart Performance Assessment and Advisor (SPA&A) section.

***3.2.6. Smart Performance Assessment and Advisor*.** The Smart2B gamification module will also enable the interaction between the end-users and the Smart2B innovations – located within the Smart2B cloud-platform, different energy and non-energy services will ensure that the users’ energy consumption patterns are optimized without hindering users’ comfort or preferences. One of the innovative services which will be provided is the Smart Performance Assessment and Advisor (SPA&A) ( Figure 10). Linked to the Smart Readiness Indicator (SRI) and methodology, where the smartness level of a building is assessed according to the building’s capabilities “to perform three key functionalities: optimize energy efficiency and overall in-use performance, adapt operations to the needs of occupants and adapt energy demand to grid signals, untapping energy flexibility” ( Ma & Verheyen, 2022). In line with the SRI methodology, “the three key functionalities are further detailed into a total set of seven impact criteria, including energy efficiency, energy flexibility and storage, comfort, convenience, health, maintenance and fault prediction, and information to occupants” ( Ma & Verheyen, 2022). In summary, “the SPA&A will provide the building users with data-driven insights in the current self-assessment smartness level of the building, suggesting qualitative improvement actions to increase the potential upgrading of the building, in line with the SRI definition, and show their economic and environmental impacts/benefits. The data-driven insights will raise awareness and nudge occupants towards energy efficient behavior and smart digital renovation direction, ultimately supporting informed investments in smart and energy-efficient technologies” ( Ma & Verheyen, 2022). SPA&A will partially automate the necessary SRI-related on-site inspections by linking the monitoring data, when available in the demonstration pilot sites, “with one or more specific services and their functionality levels, minimizing the inspection effort by an SRI assessor or even eliminate the requirement of on-site inspections” ( Ma & Verheyen, 2022). Based on literature review, interviews with experts and a stakeholder functionality survey, conducted in the scope of the Smart2B project alongside appropriate stakeholders, the projects deliverable D1.2 ( Ma & Verheyen, 2022) extensively covers the SPA&A service, detailing and contextualizing its functional requirements.

***3.2.7. Design and implementation*.** Based on the user-centred approach, central to the Smart2B concept, the design and implementation of the overall gamification module is conducted through an iterative improvement and development cycle. The developed platform prototypes (i.e., both the Figma prototypes and the first iterations of the Smart2B platform) are presented and tested by potential Smart2B end-users. The feedback provided is then incorporated in further developments, guaranteeing that the users’ needs and preferences are attended for. Tailoring the feel, navigation, and design of the Smart2B gamification module can help to trigger different motivational motives and further the users’ engagement and interaction with the Smart2B overall application. Below, in Figure 9, an iteration of the gamification module homepage design is displayed.

## 4. Conclusion

Seamlessly Integrated within the Smart2B UI, responsible for bridging the interaction between end-users and the Smart2B platform, the gamification module is responsible to promote and foster user-engagement, provide an improved user-experience and promote energy literacy among the Smart2B end-users. The conducted literature review, alongside the engagement guidelines developed within the project, enabled the careful identification of the most utilized and possibly the most effective game design elements in the context of energy-related gamified solutions for buildings. Hence, the Smart2B gamified solution transforms the every-day act of consuming electricity/energy into a game-like experience: by facing users with a series of gamified challenges, by fostering a cooperative competition environment, highlighting the achievable benefits, and by providing a learning platform to boost energy literacy, users are incited to optimize their energy-consumption patterns. The gamification module and the respective game design elements are implemented in Meteor, an open-source cross platform framework to build and deploy web, desktop, and mobile applications. A set of services will guarantee that the user is well informed and engaged with their own consumption patterns, in line with the user-centered pilar of the Smart2B project. Apace with the Smart2B gamification module development and implementation, platform tests are being conducted with groups of selected potential Smart2B users aiming at further improve the platform’s design, usability, and overall user-experience. According to the project’s work plan the prototype is to be deployed from November 2022 forward, moment where a public deliverable will describe in full detail the gamification component developed in the project’s scope.

## Data Availability

Zenodo: Smart2B Gamification module script and survey. https://doi.org/10.5281/zenodo.7292167. ( Cravinho, 2022). This project contains the following underlying data: Gamification module script and survey.pdf (script and survey for future workshop attendees to evaluate and assess the Smart2B project’s gamification module) Data are available under the terms of the Creative Commons Attribution 4.0 International license (CC-BY 4.0).

## Footnotes

^†^ Figma is a cloud-based UI designing and prototyping tool, which allows to collaborate on creating, testing, and deploying interface or product designs. Available at https://www.figma.com/.

^‡^ Meteor “is an open source and a full-stack JavaScript platform for developing modern web and mobile applications. Meteor includes a key set of technologies for building connected-client reactive applications, a build tool, and a curated set of packages from the Node.js and general JavaScript community. Meteor allows to develop in one language, JavaScript, in all environments: application server, web browser, and mobile device.” ( Santana & Fonseca, 2022).

^§^ Node.js “integrates directly with a mongoDB that serves as backends’ database” ( Santana & Fonseca, 2022).

^**^ React is resourced to build the front-end. It calls methods and subscribe to publications to sets of data hosted in the server side – the communication between server (back-end) and client (front-end) sides is made via web sockets, allowing bidirectional data transfer.
