# Energy gamification: design and development of a user interface tool to upgrade social experience and energy literacy

**DOI:** 10.12688/openreseurope.15158.2

**Published:** 2023-08-17

**Authors:** João Cravinho, Ricardo Lucas, Miguel Brito, Daniel P. Albuquerque, Uways Mithoowani, Nuno M. Mateus

**Affiliations:** 1 EDP NEW R&D, Lisbon, 2685 – 039, Portugal; 2 Sapienza Università di Roma, Piazzale Aldo Moro, Roma, 00185, Italy

**Keywords:** serious games; gamification; energy; energy efficiency; energy consumption; energy conservation; user engagement.

## Abstract

Gamification consists of applying typical elements of game-playing environments to other areas of activity. In various fields such as medicine, education, or business, gamification has been explored as an efficient vehicle to foster real-life predetermined targets or improve a real-life action's effectiveness. Amidst the current energy transition, gamification has emerged as a promising strategy to make the energy transition exciting to end-users, bridging information gaps, increasing learning, and motivating behaviour change. This study presents the design and development of a gamified solution as part of the Smart2B H2020 project. The primary objective is to create an excellent user-engagement experience while encouraging and fostering energy literacy and behaviour change. Leveraging the increasing digitalization of the energy sector, the developed gamified module will feature a user interface (UI) tool that promotes healthy competition between users, primarily driven by changes in energy consumption behaviour. The monthly and overall leader boards will translate energy savings into an in-game virtual point-based system, reinforcing the intrinsic value of energy conservation. The gamified elements and mechanisms, such as missions, interactive tasks or challenges, instant feedback, achievements, and badges, will progressively guide users in understanding their energy consumption patterns and how they can be improved. Drawing from social engineering and educational perspectives, the pilot sites within the Smart2B project will maximize user interaction and engagement to motivate real behaviour change. By highlighting the design and development aspects of our gamified solution, we aim to provide more insights into the process that was followed to create an effective and impactful tool for promoting sustainable energy consumption practices among end-users.

## Plain language summary

 Gamification, which consists in the application of elements which are part of game environments (such as points, leader boards, levels or stages, missions, and achievements) to real-life situations, bringing forth the motivational potential of game environments and enhancing and addressing real-life objectives. In the context of the Smart2B project, gamification will take shape in a virtual game environment – the Smart2B platform – where end-users will be engaged and incited to optimize their energy consumption patterns. By fostering a healthy competition based on a in-game point system, users will face different tasks and interactive challenges in order to foster energy conservation, increase energy literacy and ultimately to enhance user-engagement. Combining the energy consumption behaviour change while measuring the user engagement with the gamification mechanisms one can draw insights on the effectiveness of gamification.

## 1. Introduction

Human brains are wired in such a way that people enjoy engaging with challenges and platforms, reaping the positive feedback, rewards, and the social-bonding perspective that games provide. They are one of the most widespread strategies to which human beings’ resort to either interact, communicate, or simply have fun. With the advent of digital technology, games have become even more accessible to people – recently, video games have become increasingly popular among all ages and gender groups, often regarded as the “central entertainment media of the future” ( McGonigal, 2011). To differentiate the large number of games and video games, and to make them more compelling, current games rely heavily on user engagement mechanisms, such as points, badges, a compelling narrative and user experience, or virtual in-game currency. With the increasing capacity to generate and process data the paradigm of purely-entertainment driven games has shifted to the increase of ‘serious games’ – a non-entertainment focused game, where the primary purpose is to foster some kind of predetermined action or activity (e.g., such as improving the learning experience), instead of hedonic games ( Brackenbury & Kopf, 2022). There is no doubt about the inherent motivational potential that video games, and games in general, possess. This potential has been extensively covered in literature and explored in serious gaming ( Khan *et al.*, 2020). Expanding on the concept of resourcing to gamified elements to improve an activity’s efficiency, gamification has surfaced to bring the motivational power of video games to real-life and real-world applications. Gamification imports elements, mechanics, design, and principles of game-theory and game environments into other areas of activity, usually real-world contexts, transforming everyday real-life activities into game-like experiences ( Beck *et al.*, 2019). Since its wide adoption, from 2010 onwards, gamification has extended to pretty much all areas of human activity – from work ( Ferreira-Oliveira *et al.*, 2017), to medicine ( Sardi *et al.*, 2017), education ( Nevin *et al.*, 2014), or even within the energy sector, gamified solutions are increasingly being explored as an efficient instrument to engage with users and achieve real-life targets. Gamification is indeed deemed as providing positive effects, despite being “greatly dependent on the context in which the gamification if being implemented, as well as on the users using it” ( Hamari *et al*., 2014). Studies ( Beck *et al.*, 2017) also indicate that providing information through gamified solutions may increase its impact in comparison to common communication channels. In the context of the current energy transition and leveraging in the increasing digitalization of the energy sector, gamified solutions can provide a useful user-engagement platform while fostering energy-consumption behavioural-change. The European building stock is currently responsible for almost 40% of final energy consumption and 36% of the final CO _2_ emissions globally ( European Comission (EC) 2020). Adding to the fact that people spend a large amount of their time inside buildings and that around 75% of the current EU-27 building stock is “energy inefficient” ( Lewis *et al.*, 2021), it serves as an effective vehicle of change for the energy transition targets ( European Comission (EC), Directorate-General for Climate Action 2019). In this context, the Smart2B H2020 project ( Smart2B H2020 Project, 2022), which aims to upgrade the smartness levels of existing buildings through coordinated cloud-based (i.e., Smart2B platform) control of legacy equipment and smart appliances while offering new energy and non-energy services (e.g., increased energy efficiency, improved indoor comfort to the occupants and flexibility) to various stakeholders, will be able to provide a testbed to answer the broad research question of ‘how can the energy transition in residential buildings be leveraged by gamified solutions’.

The aim of this work is to present and detail the approach taken for the developing gamified solutions to create an excellent user-engagement experience while encouraging and fostering energy literacy and behaviour change in the project's context. The functional prototypes were developed and assessed alongside potential users to validate its adequacy and to iteratively incorporate their feedback. The developed gamified solution will comprise a user interface gamified module where a healthy competition between users will take shape – driven mainly by the user’s energy consumption behaviour and behaviour-change – and the monthly and overall leader boards will translate the energy savings achieved by each user into an in-game virtual point-based system. Other gamified elements and mechanisms such as alternate missions, interactive tasks or challenges, instant feedback, badges, and the interaction with Smart2B’s innovations will further progressively guide the user through its energy consumption patterns and how they can be improved. A social engineering and educational perspective, brought possible within the context of the pilot sites in Smart2B, will focus on the maximization of user interaction and engagement and how can these gamified solutions motivate real behaviour-change.

Section 2 details a literature review of gamification and gamified solutions applied in the energy sector’s context, in 2.1, which paves to a description of the developed gamified concept and component, in Section 3. In Section 3.1 the different and relevant game design elements considered within the context of the developed gamification component are detailed. Section 4 includes the discussion and final remarks.

## 2. Gamification: literature review

Through strategic plans such as the European Green Deal ( European Comission, 2019), the “Renovation Wave” ( European Comission, 2020), and the recent REPowerEU ( European Comission, 2022) the European Union is increasingly committed to developing a sustainable, secure, competitive, and decarbonized energy sector by 2050. To achieve such goals, special focus should be given to the building sector which accounts for almost 40% of final energy consumption and 36% of the final CO _2_ emissions ( European Comission (EC) 2020) and is among the largest end-use consumer sectors ( D'Agostino *et al*., 2017). The building sector’s energy consumption reduction may be achieved by different means such as the adoption of building energy efficiency standards, promoting building renovation or resourcing to digital and ICT solutions for building automation and response, among others ( Casals *et al.*, 2020). Findings ( Zhao *et al.*, 2017) show that along with the ever increasingly capacitating technological advances in buildings systems, inciting the end-user’s engagement and behavioural change is key. Hence, gamification can be explored as an effective mechanism to further engage end-users and ultimately foster behavioural change ( Fijnheer & van Oostendorp, 2016).

Gamification is, however, a broad term, and many frameworks for its application are available. In the present case the GMC ( Escribano & Cp, 2010) was adopted. With its comprehensive and structured approach, the GMC framework allows for the connection of player motivations with desired behaviours, ensuring effective and sustainable user engagement while offering valuable insights into understanding players’ motivations and behaviour, crucial for designing effective gamification solutions. Based on the Mechanics-Dynamics-Aesthetics (MDA) framework ( Hunicke *et al.*, 2004) and the Business Model Canvas ( Osterwalder & Pigneur, 2010), the GMC framework integrates various motivation theories and behaviour models harmoniously.

Marczewski's extension of Bartle's works further enriches the understanding of players' profiles in gamification contexts ( Marczewski, 2015). The user can be categorized based on four intrinsic motivations (RAMP): Socialisers, motivated by Relatedness; Free Spirits, motivated by Autonomy and self-expression; Achievers, motivated by Mastery; and Philanthropists, motivated by Purpose and meaning. Additionally, extrinsically motivated user types, collectively referred to as Players: Self-Seekers, Consumers, Networkers, and Exploiters, depending on their action or interaction with the player vs system. The final user category is the Disruptor user types, consisting of individuals who disrupt the system positively or negatively: Griefers (negative disruptors), Destroyers (breaking the system directly), Influencers (attempting to change the system), and Improvers (interacting to change the system for the better). However, the user types identified “are not intended as mutually exclusive types but rather profiles where a person can be motivated fitting multiple types to a various degree” ( Van der Neut *et al.*, 2022). Works such as ( Marczewski, 2015), ( Tondelo *et al.*, 2016) or ( Santos *et al.*, 2021) provide overall valuable insights and examples of game design elements which positively tap into the motivational triggers of each user type.

The works of ( Lieberoth, 2014) and ( Gurjanow *et al.*, 2019) also make an important distinction between shallow and deep gamification, in terms of the level of impact and transformation that they bring to the user experience and behaviour and to the core process that is being “gamified". Shallow gamification typically involves the addition of a “layer that is put above and on top of the core processes, without changing their essence” ( Gurjanow *et al.*, 2019). A good example of shallow gamification techniques are the classic Points, Badges or Leader boards (PBL) – they serve as a vehicle to further engage and motivate users, but they do not change the core processes themselves. On the other hand, deep gamification goes beyond the superficial enhancements – they introduce “game elements that change the core processes of the activity” ( Santos, 2015). These mechanisms aim to create a seamless and immersive gamified experience that aligns with the user's intrinsic motivations and values.

Recent works ( Mozelius, 2021) highlights that this distinction might become even more blurred, as the transition from shallow to deep gamification can be achieved via the continuous integration and blending of different game design elements (e.g., competitive elements, learning objective aligned with game objectives, introducing different levels of success instead of a simple win-lose scenarios, etc), resulting in the “Total gamification” scenario of ( Santos, 2015).

The next Section 2.1 reports previous studies which focused on gamified solutions tackling energy consumption and how can they maximize user engagement. In Section 3 the Smar2B’s gamified solution is described: in Section 3.1 a description of the project’s use case at hands, followed by a detailed description of the gamification module concept and components in 3.2. Section 3.3 provides some insights in the present study’s limitations and future works.

### 2.1. Gamification in the energy sector

By bringing the motivation enhancement aspect of game environments to the real-life demand-side energy system environment, it is possible to further address the energy transition targets within the ineffective ( Lewis *et al.*, 2021) and energy-intensive ( European Comission, 2019) European building stock, while motivating real-life behaviour change in the building’s consumption patterns and fostering energy literacy among end-users. Hence, successful gamification within the energy sector must act in two distinct fronts: incite the users’ short-term engagement, by fostering some real-life benefits which act as incentives (extrinsic motivation), without neglecting the much-needed long-term engagement, by building the intrinsic motivation, unlocking the possibility to motivate real-life energy consumption behaviour change. The diverse panoply of game design, principles, elements, and mechanisms (see Section 1) that are brought to the real-life environments constitute the building blocks of gamified solutions, as the game design elements ( Sailer *et al.*, 2017). They represent different mechanisms through which different motivational outcomes are triggered. For the engagement to succeed, these motivational outcomes must be compliant with the users’ needs and target them: the users’ behavioural constructs and psychological needs ( Frederiks *et al.*, 2016) towards energy consumption must be addressed *via* the game design elements. The literature concerning gamification applied in energy-related behaviour change within the context of the residential sector is today widely available – for example, comprehensive ( Grossberg *et al.*, 2015) and methodological reviews of pertinent projects in the topic and their main conclusions may be found in ( Pasini *et al.*, 2017), ( Johnson *et al.*, 2017), ( AlSkaif *et al.*, 2018), ( Beck *et al.*, 2019), or ( Chatzigeorgiou & Andreou, 2021) – all of them seem to point to the promising results of gamified energy saving programs when it comes to energy savings achieved, ranging from 4% to 24% ( Van der Neut *et al.*, 2022) ( Iweka *et al.*, 2019). In addition, ( Fijnheer *et al.*, 2019) showed the benefits of game environments *vs* a traditional dashboard-only approach to achieve a more engaging, sustained, and effective change in the users’ energy and gas consumption patterns.

Several previous studies in the context of other H2020 projects focused on and have demonstrated the potential of gamification in encouraging energy-saving behaviours and fostering behavioural change. These examples include ENTROPY project ( Kotsopoulos *et al.*, 2018), which propose a serious game approach, devised “a modular, rule-based mechanism for formulating personalized energy-savings recommendations and tips tailored to the users’ profiles and game design choices”, in workplace contexts. TRIBE ( TII *et al.*, 2016) also delved in serious games to motivate energy savings, in multiple contexts (residential and workplace). An independent report ( Deloitte, UNIGRAZ 2018) estimated achieving energy-savings up to 15.5% and reaching 18,977 players. In turn, GAIA project explored multiple approaches, namely serious games, the application of gamification elements in a mobile (and tablet) application and an in-person toolkit for energy awareness ( Mylonas *et al.*, 2019a). The project’s applications and web-based tools for promoting “energy awareness about energy consumption and sustainability, based on real-world sensor data (…), while also leading towards behaviour change in terms of energy efficiency” ( Mylonas *et al.*, 2019b). The real sensor data was collected in the context of public buildings such as schools.

A different approach is used by the Social Power Game ( De Luca & Castri, 2014), which consists in a mobile application designed to promote sustainable energy consumption through social interactions and gamification. By connecting neighbourhoods, the app encourages collective energy-saving practices and the adoption of sustainable lifestyles. The game featured personalized tracking of household electricity consumption, providing users with easy-to-read visualizations, as well as informing them of the impact of their actions and the individual player’s contribution to his team achievements. Players were assigned to teams upon registration and receive individual, collaborative, and cooperative challenges to earn points. Additionally, the app offered information on making more efficient use of shared resources. With leader boards and badges for achievements, the game fostered healthy competition and a sense of accomplishment. Preliminary results ( Castri *et al.*, 2016) revealed promising results, with 75% of participating households reducing their historical consumption. The game's combination of social collaboration, gamification elements, and personalized tracking proved effective in encouraging users to actively engage in energy-saving behaviours and contribute to their teams' achievements. ( Wemyss *et al.*, 2019) reported short-term energy savings achieved ranging from 7.8% to 8.5% across two groups. However, in the long term, the same study found “electricity savings achieved during the intervention were not maintained”.

Another example is the case of “Cool Choices” ( Ro *et al.*, 2017), that is was a game in which players competed as teams to reduce energy usage over a multi week period. Players claimed points in the game for engaging in either one-time or recurring sustainable behaviours. Alike the approached used in Smart2B (see Section 3), these pro-environmental actions were made visible to other players through the game’s leader board. An evaluation study revealed that playing ‘Cool Choices’ led to long-term reductions in electricity consumption, especially among individuals who initially consumed high amounts of energy ( Ro *et al.*, 2017), ( Benjamin & Brauer, 2021).

( Koroleva *et al.*, 2019) aimed to design and evaluate a holistic socio-technical behaviour change system for energy saving, incorporating insights from behavioural theories and persuasive system design. The system combined smart meter data with interactive visualizations of energy consumption, gamified incentives mechanisms (virtual and tangible rewards), energy-saving recommendations, notification, and attention triggers. In addition, the researchers conducted a real-world pilot to evaluate the effectiveness of the non-personalized energy-saving system, indicating a 5.81% of energy decrease, compared to the baseline period. Moreover, a positive change in energy-related knowledge was showed in users. The combination of behavioural theories and persuasive design elements in the system contributed to its success in promoting sustainable energy consumption practices among households. For users to better relate with energy-savings metrics, ( Melenhorst *et al.*, 2018) and ( Koroleva *et al.*, 2019) showed that it may be beneficial to apply metaphors for the three main goals of energy savings – monetary (reduce energy costs), sustainable (environmental impact) and hedonistic (taking pleasure while saving energy).

The examples presented previously highlight the effectiveness of gamification approaches in the energy sector, indicating that more personalized or tailored approaches can significantly enhance energy-savings compared to a standardized solution ( AlSkaif *et al.*, 2018). The effectiveness of the gamification approach can also be dependent on the aesthetics and quality of the design implementation ( Grossberg *et al.*, 2015) ( AlSkaif *et al.*, 2018). Studies, including ( Osbaldiston & Schott, 2012), ( Šćepanović *et al.*, 2017) or ( Chatzigeorgiou & Andreou, 2021), propose employing a combination of game design elements to enhance the effectiveness of gamified approaches. These highlight that incorporating strategies such as rewards and goals, instructions, and goals, as well as commitment and goals, can generate a more impactful gamification experience. By combining different game design elements, gamified systems can achieve improved results.

Combining different game design elements is also identified as a promising approach, as proposed in. This involves employing diverse strategies, such as incorporating rewards and goals, instructions and goals, as well as commitment and goals, to enhance the overall effectiveness of the gamified approach ( Osbaldiston & Schott, 2012).

Moreover, ( Sailer *et al.*, 2017) provides a comprehensive study and framework on the effectiveness of game design elements to address the users’ psychological and intrinsic needs in energy consumption behaviour change – studies have shown that users’ energy consumption patterns “will be enhanced when users’ needs for autonomy, competence and relatedness are supported” ( Wee & Choong, 2019).

## 3. Smart2b gamification concept

Drawing on the work within the consortium and the stakeholder framework ( Croé, 2022), research work, including interviews, was conducted to analyse and characterize the Smart2B’s group of actors – building owners, building managers, occupants, grid operators and groups of citizens, whose needs and functionalities at the platform and UI level are further discussed in the next Section 3.1. The latter two actors, however, are not a concern of focus for the developments since they’re secondary actors. Considering the diverse audience in terms of groups of actors and attending to the project’s pilots’ specific needs, which range from the residential sector in Portugal with occupants who are kids and managers and owners as the respective responsible parties, to the also residential sector (nursing homes) in Denmark, and drawing inspiration in the relevant gamification frameworks (previous Section 2) as well as past projects and initiatives, the design approach adopted was a generalist solution which would encompass as much of the players’ user types, while also satisfying their psychological needs ( Wee & Choong, 2019).

**Figure 1.**
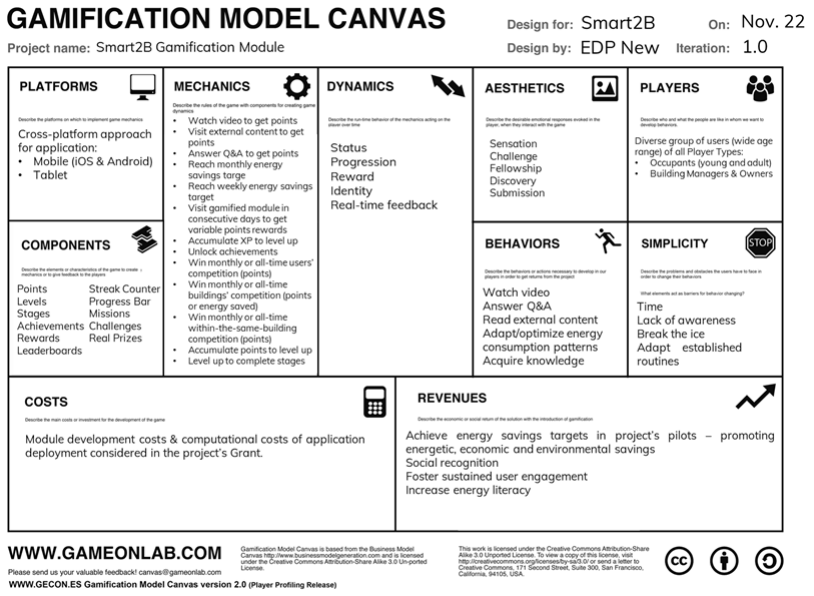
Smart2B’s project Gamification Model Canvas., adopted from ( Escribano & Cp, 2010).

Thus, the gamified solution will focus on promoting a cooperative competition environment focused on achieving energy savings and energy consumption behaviour-change among the platform’s users. Missions, smaller interactive challenges, instant feedback, and badges will translate real-life actions into an in-game experience point (XP) system, through which user’s will be ranked according to their performance. Different competitions will be fostered, aiming to tap into different motivational triggers: a competition between individual users and a competition where different occupants of the same residential building will compete against other residential ‘clusters’ (i.e., other residential buildings which can have multiple occupants). Through an increasingly challenging and progressive user experience, and the interaction with the Smart2B innovations, the users will be progressively guided through their energy consumption patterns and how they can be improved. An educational layer to the game design elements, will provide an additional interaction and engagement platform while fostering real and lasting behaviour-change. Below, in Figure 1, the current project proposal is summarized resourcing to the GMC framework’s canvas.

To frame the problem to this specific case the core process is energy consumption behaviour of the houses’ occupants (and catering to the managers and owner’s needs). The gamification module of the UI serves as an immersive and engagement platform, applying those game design elements to the core process of consuming energy. While the gamification module itself can be seen as a shallow gamification approach, the interaction between the users and the Smart2B ecosystem does grant the possibility to change the core process – its services framework, developed under the project's scope, offers non-gamified functionalities such as load scheduling, control, and automation. These capabilities can create changes in the core process of energy consumption while facilitating a deeper level of user engagement, experience, and behaviour change.

Together with the work developed within the consortium ( Croé, 2022) and leveraging the synergies between consortium partners, the modular component was conceptualized and validated through a set of functional prototypes, developed in Figma ^†^, which were then tested with potential Smart2B users. These prototypes serve the purpose of illustrating the game design elements while also assisting in generating a list of functional requirements for the actual platform development. The gamification module will be coupled and seamlessly integrated in the User-Interface (UI), being developed within the consortium for the purpose of establishing the communication channel between the Smart2B platform and its end-users, involved in the demonstration activities. The Smart2B’s application and UI are documented in ( Santana & Fonseca, 2022). Hence, along with the Smart2B’s gamified solution’s concept, the game design elements which compose the gamified solution are to be developed in the existing cross-platform framework (Meteor ^‡^) – i.e., both the back (Node.js ^§^) and front-end (React ^**^) developments required to operationalize the gamified solution.

### 3.1. Use case description

The gamified environment and the gamification module, operating within the Smart2B platform, is one of the project’s use cases, focused on bridging the information gaps between users and the Smart2B platform, services, and UI – the projects’ public deliverable ( Albuquerque *et al.*, 2022) provides a more in-depth description of the different Smart2B’s use cases. Conceived with user-centred approach, the UI will be tailored to each users’ needs – the level of interaction and automation of the UI is tailored to what “each user demands from the system” ( Albuquerque *et al.*, 2022). Through an adaptive design, the UI will automatically adjust its settings, functionalities, and level of control to cater specifically to the unique requirements of the different group of actors. For instance, building managers may have access to advanced settings or administrative controls, while occupants can’t. Moreover, the UI's responsiveness and interactivity will empower all users to further customize their experience according to their preferences. Within the detailed dashboard, users can easily toggle the visibility of tabs, open and close specific sections, and fine-tune settings to align with their individual needs and workflows. This level of flexibility ensures that each user can optimize their interaction with the system, enhancing usability and overall satisfaction. By incorporating adaptive elements and empowering users to personalize their UI, the gamification module (and the entire overall application) aims to maximize user engagement, efficiency, and overall user experience. This approach recognizes the diversity of users interacting with the system and ensures that the UI seamlessly adapts to cater to their distinct roles, preferences, and objectives, promoting a positive and efficient interaction between users and the Smart2B ecosystem.

Within the project, different actors will communicate via the dashboards and UI with the Smart2B ecosystem and platform ( Santana & Fonseca, 2022). Each group of actors, identified in ( Croé, 2022) – building managers, occupants, grid operators and (groups of) citizens – will have different application’s profiles and consequently different functionality levels within the virtual environment, ensuring that “all actors will only see relevant information for them so only the needed functionalities and data will be presented to all of them” ( Albuquerque *et al.*, 2022).

**Figure 2.**
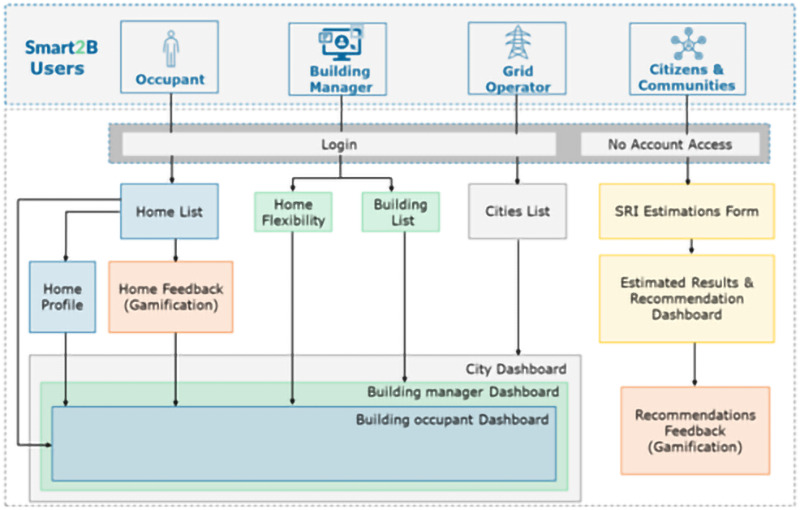
Diagram illustrating the Smart2B use-case related to the Smart2B Application, from ( Albuquerque *et al.*, 2022).

Building managers and owners will be able to access a list of all the buildings they manage or own, including the option to add new ones (and depending of its role, to also add new occupants or building managers or owners). During the process of adding a new building, users will be asked to provide data for building identification and to set the maximum and minimum values for three adjustable dimensions: comfort, energy savings, and environmental impact ( Albuquerque *et al.*, 2022). It should be noted that manipulating any of these dimensions will have an impact on the others, and “users must accept this trade-off” ( Albuquerque *et al.*, 2022). On the other hand, occupants will also have real-time access to monitor the electric energy flows (demand and generation, if present) and Smart Readiness Indicator (SRI) ^††^ level of their houses, while taking into consideration the possibility of each occupant user having more than one residency. Grid operators will be able to access and monitor the flexibility services “proposed to consumers are being used/accepted by them” ( Albuquerque *et al.*, 2022), with an on-demand granularity possibility (i.e., from cities to apartments). Citizens or groups of citizens will be able engage and access the Smart2B platform to estimate their homes/buildings SRI level. A descriptive diagram of the use case can be found in Figure 2, below. A more detailed description of the use case can be found in ( Albuquerque *et al.*, 2022).

The different game design elements within the virtual environment of the Smart2B platform, described in the upcoming section, with which the users will be able to interact will be monitored alongside the energy consumption patterns. The gamified module is accessible to all profile – occupants/owners, and managers. The association will be between user and building, inviting everyone associated with the building to participate, thus increasing the reach. The interaction level of the users with the different gamified mechanisms can then be compared with the monitoring of the end users’ energy consumption patterns to draw insights on the effectiveness of the different game design elements.

To collect user feedback regarding the gamification component’s functionalities and its overall feel and look, local workshops will take place with groups of users, belonging to the different Smart2B group of actors. A manual will be provided to the users, guiding them through the app’s virtual environment highlighting some of the key design elements (see Section 3.2), and a set of linear numeric scale and open-ended questions to evaluate their experience (see Section 3.2.7).

### 3.2. Game design elements

When users navigate through the UI to the gamification module for the first time they’ll be presented with an initial ‘Hero Page’ ( Figure 2). This page will contain an introduction to the gamification module: the clear guidelines, rules of play, and goals of the gamified mechanisms and elements which the users will face, as well as the benefits (individual *vs* collective and real-life *vs* virtual) of participating in the designed gamified solution. The highlighted information will speak to the core of the gamification concepts explored in 2.1 (e.g., the users psychological needs) while also emphasizing the different and achievable benefits, which can act as incentives to users ( Beck *et al.*, 2019) – whether it’s from an individual perspective or from a community point-of-view the economic, environmental, or social incentives and benefits can be tapped and enhanced by gamified solutions ( AlSkaif *et al.*, 2018).

**Figure 3.**
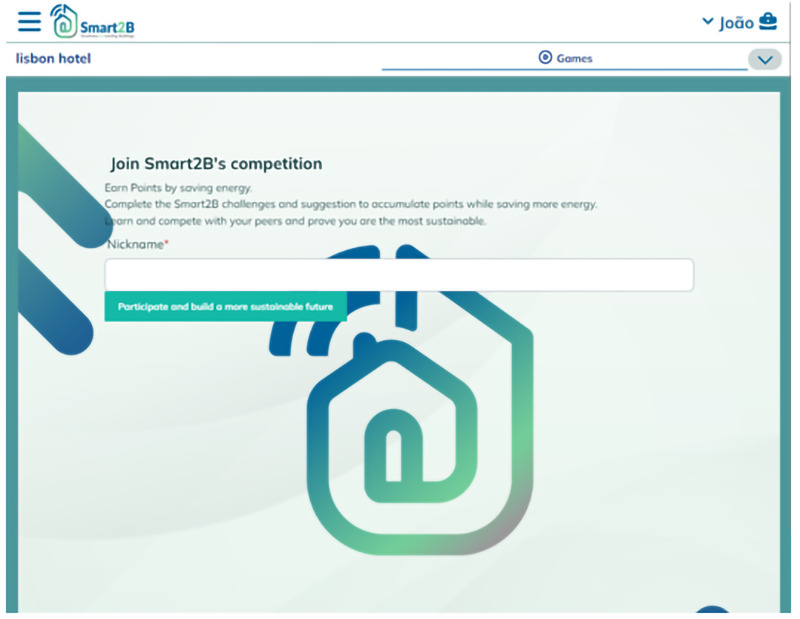
Meteor prototype of the introductory (Hero) page displayed to first-time users of the gamification module.

**Figure 4.**
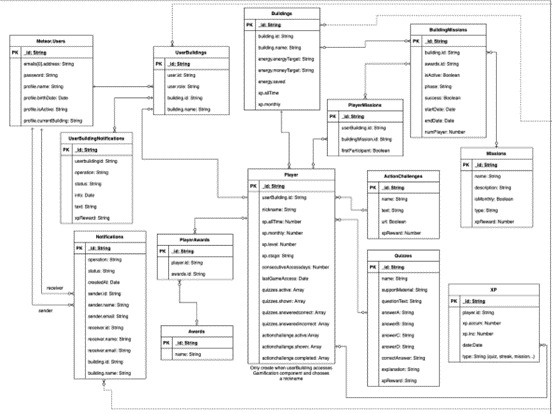
Back-end data model for the Smart2B user interface (UI) and gamification components.

Upon clicking the displayed button (in Figure 3, represented by the green ‘Participate’ button), a game profile will be created for the user – a mongoDB data collection, automatically generated for each new player (i.e., an association between a user and a building). The player profile will figure every back-end piece of information, data, or variables associated with the user, and associated building, which needs to be transferred among game design elements (e.g., users’ unique identification, the building’s unique identification, the amount of points the user accumulated so far, etc), ensuring consistency in the data model throughout the different game design elements implemented. In Figure 4, the data model structure for the gamification module and UI is presented. The data model, alike relational databases, is composed of tables and relationships between them through primary keys, PK, avoiding data overlapping and duplication – each table groups different variables of the same nature.

**Figure 5.**
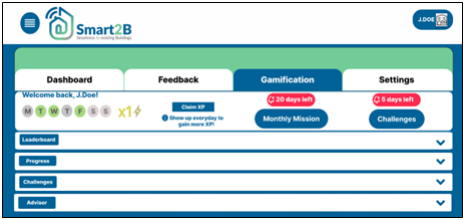
Figma prototype of the gamification module homepage. XP stands for experience points.

Consequently, the user will be forwarded to the gamification module homepage as shown in Figure 5. In the next sections the main game design elements included in the Smart2B’s gamification module are detailed.

**Figure 6.**
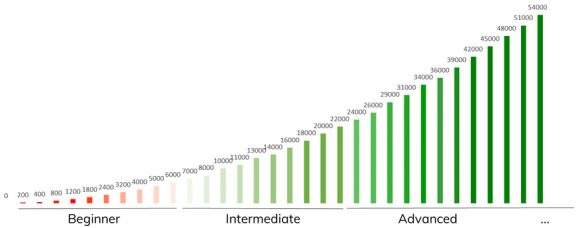
Schematic representation of the Smart2B gamified journey: each experience stage (Beginner, Intermediate and Advances, below) is composed of 10 intermediate levels. Each bar represents one level, displaying above the accumulated XP points required to reach it.

***3.2.1. In-game point system*.** The designed in-game environment point system is the main building block of the gamification concept. Points are to be rewarded to a user as an in-game consequence of successfully completing a task, request, or to reward engagement. The experience point (XP) system is composed by two main components, levels, and stages, creating an incremental and progressive environment. The schematic representation of the designed experience point system is shown in Figure 6.

in line with the approach followed by ( Amy, 2012) Player’s Journey, there are three stages, which represent big milestones for the user:

▪ Beginner: the user begins its journey on the first stage, the ‘beginner’ stage, where they must learn how to work with the application basic features. Here it is recommended to provide initial information to the platform, as well as provide feedback regarding equipment usage, preference settings, among others. These actions ensure that the player is comfortable with the whole gamification module and platform.
▪ Intermediate: after gaining 6000 XP, which should translate into around six months of consistent usage, the user ‘levels up’ to the second stage, ‘intermediate’. Here they should be a knowledgeable user of the platform and are encouraged to improve their energy saving with weekly or daily goals. At the same time, an increase in knowledge around energy-related topics is facilitated with the help of quizzes, topical questions and informative videos (see the educational challenges, described under Section 3.2).
▪ Advanced/Ambassador: at 24000 XP, approximately 18 months of app usage, the user arrives at the third and final stage, where they are a proficient energy saver. At this stage, the user is still encouraged to improve the energy efficiency of the household while deep diving more seriously into related energy topics. It is expected, that at this stage, the user no longer needs the gamification component of the application to ensure that they maintain their behaviour, therefore this component has had the desired effect of instilling the intrinsic behaviour which promotes energy savings, (practical and non-practical) sustainability-driven actions, and knowledge.

The other experience point system gamified mechanism are the levels. The levels give the user an incremental sense of growth and improvement in the platform. Operating in a smaller scale than the stages, the levels are more easily attainable and achieved, keeping the user engaged. Each stage is divided by 10 levels with incremental gaps of XP to ensure that the user feels a continuous experience throughout the user experience, with the exception of the third stage (Advanced) which has no limit to the number of levels, ensuring that the game does not come to an end and that the users will have the continuous experience until the end of the project’s demonstration actions.

Two different entities can ‘earn’ experience points: the user and the building, in which the users live or work. Buildings XP can only be obtained through challenges directly related to energy savings, while the user is also encouraged to strengthen their knowledge and give feedback to the platform by earning experience points from all gamified challenges (see Section 3.2).

**Figure 7.**
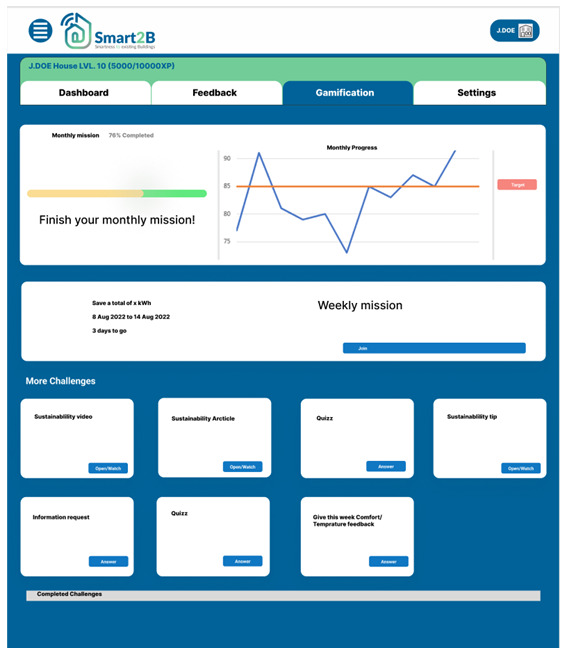
Figma prototype of challenges section of the Smart2B gamification module.

***3.2.2. Gamified challenges*.** Anchored and leveraged by the in-game experience point (XP) system described above, users will be faced with interactive challenges which tap into different motivational triggers or incentives. Five different gamified challenges are considered: missions, information requests, quizzes, videos and articles, each rendering different points to the user. Figure 7, displays a prototype of the challenges section of the gamification module.

The user will be able to see the various gamified activities, challenges, and missions which will further address and contribute to the short and long-term engagement while addressing different motivational triggers. Despite the prototype, displayed in Figure 7, showing all types of challenges, only four at a time will be displayed to the user. It is possible to categorize the challenges by their main theme:

○ Missions: Two missions are to be considered. The main monthly mission, worth 200 points, is always related to lowering energy consumption compared with the previous month’s consumption. This mission increases in difficulty to keep up with the user level of expertise and knowledge. The main objective of this monthly task is to keep the user focused on the topic of lowering energy consumption as the main goal of the gamification application, without hindering the users’ comfort level. The weekly mission, similar to the previous, focuses solely on the energy consumption of the user. Through various prompts such as minimizing consumption for a day below a certain value, minimizing overall weekly consumption or establishing a comparison between users’ energy consumption metrics, this weekly challenge makes sure that the user can feel the benefits of saving energy not only at the end of the month, but on a weekly basis. This challenge will be worth 75 XP, with a bonus of 50 XP for the first user to join the mission. The main, monthly, mission will run through each month – since the first day of the month until the day before the end of the month –, while the weekly mission will run from every Monday to each Saturday morning, giving time to every user to be aware of the next week’s mission content, speaking to the challenges’ discrete timeline guideline, as described in the previous Section 2.1.

The remaining challenges will be composed by three rotating weekly challenges which will more heavily depend on the user level, in order to guide them through the platform at a suitable pace, not letting the user feel overwhelmed at the beginning of the experience or eventually leading to boredom with a lack of tasks to perform. These side missions will be worth from around 20 to 40 XP, depending on difficulty, to which certain bonus might be added. We can distinguish two types of challenges:

○ User focused challenges: Tasks like navigating and displaying specific information within the Smart2B platform, especially during the beginning of the demonstration activities, such as energy consumption, generation, or flexibility, belong under this category. The user is encouraged to continue to provide feedback throughout the project – relative room temperature and humidity are some of the data required. These tasks will reward the user with 25 XP.

○ Instructional challenges: The last type of missions will be mostly informative and educational, this includes both quizzes and single questions, as well as informative videos for the users to learn more about certain topics. These tasks are more time consuming and involve a more active participation by the user, therefore the successful accomplishment will be reward 40 XP.

Additionally, the user can also be attributed a bonus of 50 experience points by completing all four weekly challenges (with the exception of the main monthly mission), making sure that they are encouraged to keep completing tasks after the monthly mission or even if the tasks have a higher degree of complexity.

***3.2.3. Leader boards*.** The goal of this section is to let the user know how they compare with the overall population that is also playing the game and giving them a goal to strive for, bringing the motivational triggers to real-life and inciting users to improve their experience points by completing challenges and to improve their household by saving more energy, furthering user-engagement. The leader boards present player name, current stage, current level and XP or the building’s normalized energy consumption (kWh/m ^2^). The goal is for users to strive to be top of both the overall ranking, as well as the monthly one, either by themselves or with their building (energy-related and progress-related), to accrue more XP.

**Figure 8.**
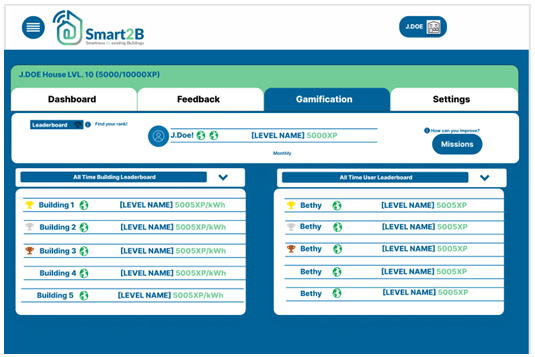
Figma prototype of leader boards section of the Smart2B gamification module.

This section is divided in two main leader boards, one which is focused on the user and one focused on the buildings. In the building leader board, only buildings are compared with each other, either by XP gained from the monthly and weekly mission only, or by their normalized energy consumption, in the form of kWh/m ^2^. The building competition serves the purpose of creating a common goal for all occupants of the same building and to compete with other buildings. The user leader board is used to compare the XP accumulated by the users, with an option to compare yourself to all users participating in the competition or to the other Smart2B platform users which are associated to the same building, inciting a friendly competition between users in the same conditions. Both classifications have the option to see the all-time comparison between members or to just compare a single month. This option allows the user to see the progress made in each month. Figure 8 shows the prototype for the web section, illustrating the different functionalities of the leader boards and the different layers of competitions.

***3.2.4. Engagement and rewards*.** The user is incentivized and rewarded for continuously interacting with the Smart2B gamification module – by completing tasks, accessing every day or by the continuous improvement made throughout the user journey. Besides the bonus rewards described in 3.2, in which the user can accumulate bonus points if all weekly challenges are successfully completed and a bonus XP for the first user who subscribes to the weekly mission, different engagement and rewarding mechanisms are contained within the Smart2B gamified solution:

Firstly, a ‘login’ streak counter informs and rewards users for continuously accessing the gamified module. For each consecutive day the user accesses the platform, the user accumulates additional five XP. By accessing the module in consecutive days, the user will be rewarded with a bonus of XP points, proportional to the number of consecutive days they have accessed the gamified module. By accessing two days in a row a user will be awarded five XP points, while at the seventh consecutive day the bonus increases to 50.

Secondly, an end of month bonus rewards the user for all the improvement made during that timeframe. Throughout the month, the user’s saved energy and process through the leader boards is calculated and, along with the earned experience points, earn the user a monthly bonus. This bonus depends on leader board position in terms saved energy, in kWh, and on the experience (XP) points achieved during that month: for every 100 XP won during the month, the user gains an extra five experience points.

Finally, badges will not render any XP to the users, and they are awarded to signal certain achievements or milestones. Finishing missions, saving a certain amount of energy (Wh or kWh), successfully completing challenges, being the first in the leader board, among others, are all ways of earning badges that represent the user’s achievements and improvements throughout the gamification component’s journey.

**Figure 9.**
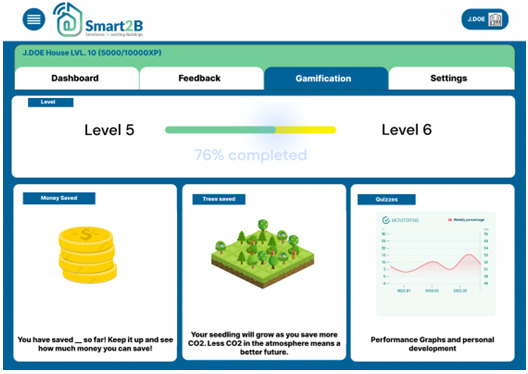
Figma prototype of progress section of the Smart2B gamification module.

***3.2.5. Progress*.** The information feedback loop, crucial to keeping users engaged, will take shape within the progress section. This section gives easily accessible information to the user about its improvement and achievements. Here the user can see information about the current level and points accumulated, the amount of energy and money saved and the progress throughout the challenges which the user is faced with. This section of the component focuses ( Figure 9) on providing the intrinsic reward and motivation to the user, through three different incentives, each with the focus to show users the benefits the user has been able to achieve during its participation in Smart2B’s gamified competition: monetary savings, emissions savings and personal development:

▪ Personal development: the user can see how much XP they have earned so far, as well as how much XP is left for the next level and stage. The information feedback will relate to the overall progress of the user since the beginning of usage of the gamification component in terms of accumulated XP. This will reinforce positive feedback to the user, inciting a continuous use of the application.

▪ Monetary incentives: a conversion from energy saved into money saved is considered. Here, depending on the energy tariffs the user benefits from, it can be seen the monetary benefits of using smart appliances and managing energy consumption. Monetary benefits can be a big persuasive for users to implement new tasks and improving the smartness of the building.

▪ Emissions savings: the energy saved is converted into saved trees. A CO _2_ to trees convertor will be used to let the user know how much they are helping the atmosphere and the whole planet. Similarly, to the monetary benefits, the environmentally friendly mentality is a good one to try to implement on our userbase, which might in the future lead them to adapt more environmentally friendly practices.

**Figure 10.**
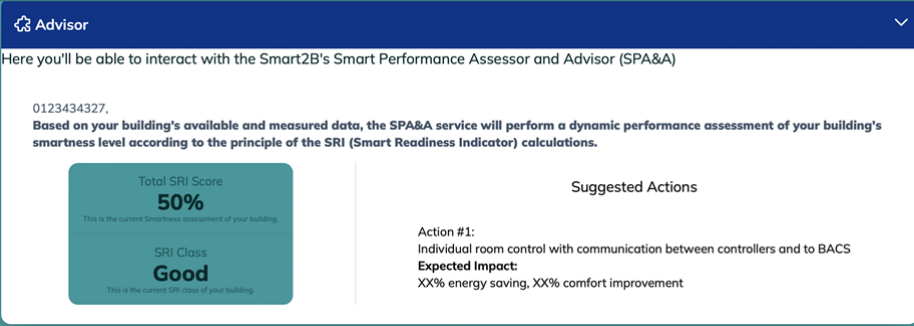
Meteor prototype of the Smart Performance Assessment and Advisor (SPA&A) section.

***3.2.6. Smart Performance Assessment and Advisor*.** The Smart2B gamification module will also enable the interaction between the end-users and the Smart2B innovations – located within the Smart2B cloud-platform, different energy and non-energy services will ensure that the users’ energy consumption patterns are optimized without hindering users’ comfort or preferences. One of the innovative services which will be provided is the Smart Performance Assessment and Advisor (SPA&A). Linked to the Smart Readiness Indicator (SRI) and methodology, where the smartness level of a building is assessed according to the building’s capabilities “to perform three key functionalities: optimize energy efficiency and overall in-use performance, adapt operations to the needs of occupants and adapt energy demand to grid signals, untapping energy flexibility” ( Ma & Verheyen, 2022). In line with the SRI methodology, “the three key functionalities are further detailed into a total set of seven impact criteria, including energy efficiency, energy flexibility and storage, comfort, convenience, health, maintenance and fault prediction, and information to occupants” ( Ma & Verheyen, 2022). In summary, “the SPA&A will provide the building users with data-driven insights in the current self-assessment smartness level of the building, suggesting qualitative improvement actions to increase the potential upgrading of the building, in line with the SRI definition, and show their economic and environmental impacts/benefits. The data-driven insights will raise awareness and nudge occupants towards energy efficient behaviour and smart digital renovation direction, ultimately supporting informed investments in smart and energy-efficient technologies” ( Ma & Verheyen, 2022) - see example in Figure 10, below. SPA&A will partially automate the necessary SRI-related on-site inspections by linking the monitoring data, when available in the demonstration pilot sites, “with one or more specific services and their functionality levels, minimizing the inspection effort by an SRI assessor or even eliminate the requirement of on-site inspections” ( Ma & Verheyen, 2022). Based on literature review, interviews with experts and a stakeholder functionality survey, conducted in the scope of the Smart2B project alongside appropriate stakeholders, the projects deliverable D1.2 ( Ma & Verheyen, 2022) extensively covers the SPA&A service, detailing and contextualizing its functional requirements.

**Figure 11.**
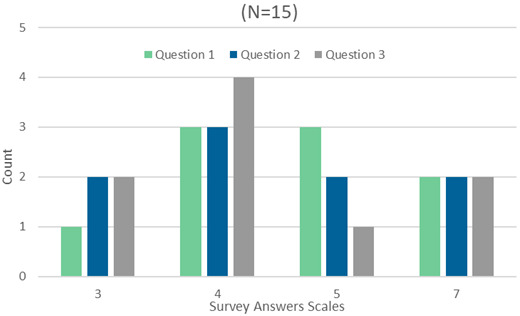
Summary of gamification module assessment survey answers.

***3.2.7. Design and implementation*.** Feedback from the gamification module, encompassing its functionalities, overall look and feel, and user experience, is being iteratively and progressively collected through a combination of methods. Dedicated testing sessions with potential Smart2B users have been conducted in local workshops, and additional feedback is being gathered via online forms. The data collected through these channels will be presented and reported on to provide insights and improvements for the gamification module based on the user's experiences and preferences. Additionally, online tests of the entire Smart2B application (including the gamification module) can be carried out through a web link and disseminated by the project partners (e.g., general assemblies of the project). Alongside the application’s web-link, users are presented with a manual, guiding them through the gamification module while highlighting its key game design elements. Adopting the Usability Metric for User Experience-Lite (UMUX-Lite) ( Sauro, 2017), users were presented with a set of three linear numeric scales (where 1 corresponds to minimum rating and 7 to maximum the rating) which assessed the tasks users faced within the manual, as well as their experience in the virtual gamified environment. Additionally, one open-ended question was included for users to provide suggestions and comments regarding the application, its functionalities and how could they be improved. Below, the survey’s questions and answers (see Figure 11) are summarized respectively – the survey and manual can also be found in the Data availability section, below.

▪ Question 1: “The several elements found within the Gamification component (point system and how can you accumulate more points, competitions, and leader boards, challenges, etc) and its rules-of-play are clear to the user?”

▪ Question 2: “Users can easily travel through the gamification module pages and identify the displayed information?”

▪ Question 3: “Users can easily travel through the gamification module pages and identify the displayed information?”

A total of 15 different surveys were collected either during the first local dedicated workshop, or via online form. The group of actors covered were Occupants, Building Managers and Owners. The average age of the survey replies was 23 years. Question 1 averaged 5.3 (out of 7), question 2 averaged 5.1 (out of 7) while question 3 averaged 5.1 (out of 7). Through the high correlation between UMUX-Lite and SUS it’s possible to estimate the second ( Sauro, 2017) – hence, the users’ assessments of the gamification module was of 64.86 (out of 100), indicating an “ok” usability: above “poor” but still not “good” ( Sauro, 2011). The SUS score achieved shows that the gamification module is marginally acceptable with a clear possibility and need to further improve – specifically, the component’s adequacy, its capacity of communicating relevant information and the overall user experience.

In the open-ended question of the survey, “users suggested that the design’s appeal could be improved (e.g., the go back button is not sufficiently highlighted, colour schemes used within the elements could be used to indicate progress status through a traffic-light approach); that the overall module could transmit its information in a more clear and understandable fashion (e.g., include units in the graphs, include a help section to display a detailed description of the rules of the game). One answer emphasized the need to have a wide range of interactive challenges (e.g., “games”, quizzes, or external content)” ( Cravinho & Brito, 2022).

### 3.3. Future works and limitations

The present approach gamified solution presents some limitations that warrant consideration for future work. Firstly, the approach taken is generalist, aiming to cater to a wide range of users and accommodate various actors within the Smart2B platform. While this inclusivity is advantageous, it may raise concerns about the effectiveness of the gamification elements for each user group. Therefore, future research should focus on tailoring the gamified solution to specific user profiles and needs. Additionally, the integration of the taxonomy of player types as a basis for gamification design elements requires validation to ensure that the chosen game mechanics resonate with the targeted users and drive desired behaviours. To enhance the assessment of the gamified approach, more in-depth evaluation methods, such as more extensive questionnaires or user interviews, should be incorporated to collect detailed feedback from end-users. This feedback can then be used to iteratively improve the gamification module, making it more engaging and impactful in fostering energy literacy and behaviour change. Furthermore, future works may include extending the gamification module to other non-gamified functionalities of the Smart2B ecosystem, such as actuation. By integrating gamification elements into these aspects of the platform, users can be further incentivized to actively engage with energy-saving actions and real-time energy management. This expansion would provide a more holistic gamified experience, encouraging users to not only be aware of their energy consumption patterns but also take direct actions to optimize energy usage in their buildings. Moreover, incorporating elements of actuation within the gamification module could potentially lead to even greater energy savings and a more significant impact on energy literacy and sustainability awareness. By continually refining and iterating on the gamified solution, we can create a more impactful and sustainable approach to promoting energy literacy and contributing to a more sustainable future.

## 4. Conclusion

Seamlessly Integrated within the Smart2B UI, responsible for bridging the interaction between end-users and the Smart2B platform, the gamification module is responsible to promote and foster user-engagement, provide an improved user-experience and promote energy literacy among the Smart2B end-users. The conducted literature review, alongside the engagement guidelines developed within the project, enabled the careful identification of the most utilized and possibly the most effective game design elements in the context of energy-related gamified solutions for buildings. Hence, the Smart2B gamified solution transforms the every-day act of consuming electricity/energy into a game-like experience: by facing users with a series of gamified challenges, by fostering a cooperative competition environment, highlighting the achievable benefits, and by providing a learning platform to boost energy literacy, users are incited to optimize their energy-consumption patterns. The gamification module and the respective game design elements are implemented in Meteor, an open-source cross platform framework to build and deploy web, desktop, and mobile applications. A set of services will guarantee that the user is well informed and engaged with their own consumption patterns, in line with the user-centred pilar of the Smart2B project. Apace with the Smart2B gamification module development and implementation, platform tests are being conducted with groups of selected potential Smart2B users aiming at further improve the platform’s design, usability, and overall user-experience. According to the project’s work plan the prototype is to be deployed from November 2022 forward, moment where a public deliverable will describe in full detail the gamification component developed in the project’s scope. Future works may have to validate some assumptions regarding the target audience and the gamification module’s adequacy, despite the initial positive feedback gathered.

## Data Availability

Repository: Smart2B – Gamification module script and survey. https://doi.org/10.5281/zenodo.8214727 This project contains the following underlying data: Smart2B – Gamification module script and survey.pdf (script and survey for workshop attendees to evaluate and assess the Smart2B project’s gamification module) Data are available under the terms of the Creative Commons Zero “No rights reserved” data waiver (CC0 1.0 Public domain dedication).

## Footnotes

^†^ Figma is a cloud-based UI designing and prototyping tool, which allows to collaborate on creating, testing, and deploying interface or product designs. Available at https://www.figma.com/.

^‡^ Meteor “is an open source and a full-stack JavaScript platform for developing modern web and mobile applications. Meteor includes a key set of technologies for building connected-client reactive applications, a build tool, and a curated set of packages from the Node.js and general JavaScript community. Meteor allows to develop in one language, JavaScript, in all environments: application server, web browser, and mobile device.” ( Santana & Fonseca, 2022).

^§^ Node.js “integrates directly with a mongoDB that serves as backends’ database” ( Santana & Fonseca, 2022).

^**^ React is resourced to build the front-end. It calls methods and subscribe to publications to sets of data hosted in the server side – the communication between server (back-end) and client (front-end) sides is made via web sockets, allowing bidirectional data transfer.

^††^ Smart Readiness Indicator (SRI) is a metric used to assess the capability of a building to effectively interact with smart technologies and systems. It measures the building's readiness to optimize its energy consumption, comfort levels, and overall efficiency through the integration of smart technologies and devices. ( Ma & Verheyen, 2022). For more details regarding the SRI methodology, see Section 3.2.6.
