# Supplementary material for: Energy gamification: design and development of a user interface tool to upgrade social experience and energy literacy
Source: Open Res Eur. 2023 Aug 17;2:130. Originally published 2022 Nov 28. [Version 2] doi: 10.12688/openreseurope.15158.2 (PMC10521033; doi:10.12688/openreseurope.15158.2)
Supplement: Peer review history

# Reviewer report: Reviewer response for version 2

**DOI:** 10.21956/openreseurope.17800.r34511

**Published:** 2023-10-06

**Authors:** Georgios Mylonas

**Affiliations:** 1 Industrial Systems Institute, Athena Research and Innovation Center, Patras, Greece

I would first like to thank the authors for their reply to the reviewers' comments and the revised text, that have led to an improved version of this paper. However, although a number of changes have been integrated to the text, I think further revisions are required before the paper is ready for publication.

 As a first quick (but important) comment, the figures of the paper still need to be revised in some cases and are currently of low quality - this could be due to compression of the PDF, but in any case, the authors need to provide figures of better quality (e.g., Figure 4 is unreadable currently) - this applies to all the figures in the text.

 Regarding my previous comment about the potential end-users and their roles, although the authors have improved the text regarding this aspect, there is still some fuzziness in their description and I still think that this aspect needs to be better described. Please fix this.

 Regarding the gamification aspects, although now there are some more details provided, I also think that this could be improved:

- please elaborate a bit more on competition between specific users and groups of people. Why have you made this choice, in which parts of the experience, what was the reasoning for providing both aspects, etc.
- please state your goals a bit better wrt. e.g. time dedicated to participate in the gamified part. How long did you intend for each session to last, how this has shaped the design, and so forth.

Regarding section 3.2.7, it is an improvement to include some user feedback, and thanks for providing this update, but it is still a bit limited. Also, what does SUS refer to? ("high correlation between UMUX-Lite and SUS", the SUS acronym is not defined previously). I would also like to see some questions regarding the actual purposes of the tool, and not just its usability. E.g., why weren't any questions related as to whether the users believe/think they can find some interesting data via this tool, or their beliefs towards sustainability, etc. Also, the average age of the respondents seems a bit low for the intended audience of the tool. How was the selection of the participants made? This is not mentioned at all in the paper.

 As a final comment, I would like to thank the authors for including references to the work done in the GAIA project; I would recommend to remove the reference (Mylonas et al., 2019b) and replace it with a reference to a much more recent and related paper (Mylonas et al.: Playful interventions for sustainability awareness in educational environments: A longitudinal, large-scale study in three countries). That work provides a discussion of the results of the evaluation of a gamified intervention for sustainability awareness in schools, that shares a number of similarities with the one discussed here. The evaluation was performed with over 3000 end-users and can provide useful insights as to what options the authors could consider for their own work in the future. It could also provide some clues with respect to aspect that could be included in this paper as well; e.g., since no evaluation results are included in this paper, the authors could provide some additional details regarding the dimensions they will be monitoring to evaluate their solution, and what they would consider as a success in their case.

 For the above reasons, I suggest that the authors revise their text.

Is the rationale for developing the new method (or application) clearly explained?

Partly

Is the description of the method technically sound?

Partly

Are the conclusions about the method and its performance adequately supported by the findings presented in the article?

Partly

If any results are presented, are all the source data underlying the results available to ensure full reproducibility?

Yes

Are sufficient details provided to allow replication of the method development and its use by others?

No

Reviewer Expertise:

Internet of Things, Smart Cities, Sustainability, Energy Efficiency, Distributed Systems, Pervasive Computing, Gamification, AR/VR

I confirm that I have read this submission and believe that I have an appropriate level of expertise to confirm that it is of an acceptable scientific standard, however I have significant reservations, as outlined above.

## References

[1] : Playful interventions for sustainability awareness in educational environments: A longitudinal, large-scale study in three countries. *International Journal of Child-Computer Interaction* .2023;35: 10.1016/j.ijcci.2022.100562 10.1016/j.ijcci.2022.100562

# Reviewer report: Reviewer response for version 2

**DOI:** 10.21956/openreseurope.17800.r34513

**Published:** 2023-10-06

**Authors:** Inma Rodriguez

**Affiliations:** 1 Department of Mathematics and Computer Science, UBICS Research Institute, Barcelona, Spain

Although the methodology and the related work have improved in this version, the evaluation is not mature enough. Authors present an evaluation of usability instead of evaluating how gamification impacts on the user experience (using gamification metrics). Note that questions 2 and 3 of the UMUX-lite are repeated and the graph with results is not well presented (only with scales 3, 4, 5 and 7). Moreover, it seems that responses do not sum up 15.

Is the rationale for developing the new method (or application) clearly explained?

Yes

Is the description of the method technically sound?

Yes

Are the conclusions about the method and its performance adequately supported by the findings presented in the article?

Partly

If any results are presented, are all the source data underlying the results available to ensure full reproducibility?

No

Are sufficient details provided to allow replication of the method development and its use by others?

Partly

Reviewer Expertise:

HCI design and methods, Gamification and game-based learning

I confirm that I have read this submission and believe that I have an appropriate level of expertise to confirm that it is of an acceptable scientific standard, however I have significant reservations, as outlined above.

# Reviewer report: Reviewer response for version 2

**DOI:** 10.21956/openreseurope.17800.r34510

**Published:** 2023-09-07

**Authors:** Georgios Pappas

**Affiliations:** 1 Lab of Educational Material and Methodology, Open University of Cyprus, Nicosia, Cyprus

In this version, the authors have proceeded with the neccessary changes. Most importantly, they provided additional content including preliminary results of a survey. Although the sample is relatively small, at this level is sufficient to get the first insights. I believe that this manuscript is ready for indexing

Is the rationale for developing the new method (or application) clearly explained?

Yes

Is the description of the method technically sound?

Yes

Are the conclusions about the method and its performance adequately supported by the findings presented in the article?

Partly

If any results are presented, are all the source data underlying the results available to ensure full reproducibility?

Partly

Are sufficient details provided to allow replication of the method development and its use by others?

Yes

Reviewer Expertise:

Gamification, Simulation, XR

I confirm that I have read this submission and believe that I have an appropriate level of expertise to confirm that it is of an acceptable scientific standard.

# Reviewer report: Reviewer response for version 2

**DOI:** 10.21956/openreseurope.17800.r34512

**Published:** 2023-08-29

**Authors:** Carlos Vaz De Carvalho

**Affiliations:** 1 Instituto Superior de Engenharia do Porto, Porto, Portugal

The article has been slightly improved, some background information about underlying frameworks has been added and marginally connected to the ongoing work.

 Unfortunately a major issue has not been solved yet which is the validation of the impact of the gamification approach in the users. We get some info about usability testing (including a rather low SUS score) but we don't get any evidence that proves that the work done is effectively contributing to the motivation of the end-users. Without that information the article doesn't add much to the field.

Is the rationale for developing the new method (or application) clearly explained?

Yes

Is the description of the method technically sound?

Partly

Are the conclusions about the method and its performance adequately supported by the findings presented in the article?

Partly

If any results are presented, are all the source data underlying the results available to ensure full reproducibility?

No source data required

Are sufficient details provided to allow replication of the method development and its use by others?

Yes

Reviewer Expertise:

Games, Serious Games, Gamification

I confirm that I have read this submission and believe that I have an appropriate level of expertise to confirm that it is of an acceptable scientific standard, however I have significant reservations, as outlined above.

# Reviewer report: Reviewer response for version 1

**DOI:** 10.21956/openreseurope.16396.r32047

**Published:** 2023-07-31

**Authors:** Georgios Pappas

**Affiliations:** 1 Lab of Educational Material and Methodology, Open University of Cyprus, Nicosia, Cyprus

The article describes the really important matter of energy literacy through gamification. The authors have developed an application based on JavaScript (Meteor, ReactJS etc) that would help users understand more on energy efficiency by using a web-based dashboard and completing missions.

 Having worked in the field (gamification + energy), the manuscript is well written and the methods of the design and development of the tool is substantially discussed.

 The tool is mainly a UI that offers some gamification elements (scoring systems, XP points etc).

 My only concern is that the tool seems to be more of an interactive dashboard and less of a game. So, I am not sure about the user engagement factor. This has to be a part of further research in a more extensive questionnaire (more detailed than the one provided at the end). Also, the performance of the tool needs to be tested in the future.

 All in all, authors did a good work with much potential.

Is the rationale for developing the new method (or application) clearly explained?

Yes

Is the description of the method technically sound?

Yes

Are the conclusions about the method and its performance adequately supported by the findings presented in the article?

Partly

If any results are presented, are all the source data underlying the results available to ensure full reproducibility?

Partly

Are sufficient details provided to allow replication of the method development and its use by others?

Yes

Reviewer Expertise:

Gamification, Simulation, XR

I confirm that I have read this submission and believe that I have an appropriate level of expertise to confirm that it is of an acceptable scientific standard, however I have significant reservations, as outlined above.

# Response

**Published:** 2023-08-04

**Authors:** João Cravinho

**Affiliations:** EDP NEW, Portugal

Dear Georgios Pappas,

 Thank you for your valuable feedback and constructive comments on our manuscript. We appreciate your insights and have made improvements to address the questions and comments raised.

 We agree that the gamified module serves as a UI enhancement for the existing application, which includes interactive dashboards and other functionalities. The purpose of the gamified layer is to provide a more engaging and immersive experience for the users while they learn about energy efficiency and sustainability.

 Concerning user engagement, we acknowledge the importance of further research in this area. While the initial feedback collected from users is now present in section 3.2.7, we understand the need for a more extensive questionnaire and performance testing in the future. Hence, we have extended section 3, which includes a discussion of the present study's limitations and potential areas for future work (section 3.3).

 We believe that addressing these limitations and conducting further research will enhance the tool's potential and effectiveness. Thank you once again for your valuable feedback, and we are glad that you found our work to have much potential. We will continue to work on improving and refining the gamified tool to better serve the needs of users and promote energy literacy.

 Best regards, João Cravinho

# Reviewer report: Reviewer response for version 1

**DOI:** 10.21956/openreseurope.16396.r31812

**Published:** 2023-07-31

**Authors:** Georgios Mylonas

**Affiliations:** 1 Industrial Systems Institute, Athena Research and Innovation Center, Patras, Greece

This paper provides an overview of the design and development of a web-based UI for a gamified solution for upgrading energy literacy within the Smart2B H2020 project. Energy literacy and sustainability awareness are very important issues as we are all experiencing currently the impact of climate change in our everyday lives.

 Although the paper is quite interesting and there are elements in it from which the community can benefit, there are two basic elements missing in the paper in my opinion:

1. Why the authors took the design decisions they present in the paper: were there any interviews held with stakeholders? What kind of processes were followed to come up with this design? This is covered a bit superficially in the paper.
2. No results are included in the paper, e.g., no end-user/stakeholder evaluation results are provided by the authors, setting aside that no results are provided about whether the solution actually achieved its results in terms of energy literacy.

The authors in this work essentially present the design of their solution, which should be backed up with more insights on the process that was followed to develop this solution. This should probably also be reflected in the title of the article, i.e., the authors could consider changing it to "Energy gamification: the design and development...". The abstract could also be updated to reflect the content of the text a bit better, in this sense.

 I also think that the paragraph in the introduction "The aim of this study... behaviour-change" should probably be updated. The text here does not delve deep enough into the issues of how gamified solutions can be engaging and effective, since it does not include end-user evaluation aspects. It is also not clear from the abstract and the introduction what is the intended audience for this solution, e.g., is it the residential or the non-residential sector, etc. The authors could identify their target groups a bit more clearly to place their research in a more definitive manner.

 In this context, there are a number of papers from other recent H2020 projects that have dealt with the issue of the actual evaluation and the results of gamified solutions for energy saving, energy literacy and sustainability awareness that are included in the related work section and could provide some needed context, since they also addressed very diverse groups (public buildings, schools, universities, social housing, office spaces, etc.). Although a reference to the results of EnergAware is included, maybe the authors could also look into results by H2020 projects like ENTROPY, TRIBE, CHARGED, GREENSOUL, GAIA and could include comparisons as to how the approach taken in this work differs or resembles the respective approaches there, e.g., in terms of game mechanics, overall design, end-user groups, etc. There are also papers that provide an overview of the recent bibliography within the area that could be added in the related work section.

 In the context of the use cases in the project, although a brief description is included in the text and a reference is made to D1.4 of the project, I think it would benefit the text to include a more complete description. Also, a number of different user groups are mentioned (occupants, building managers, etc.) in the text, without explaining much about their roles. Figure 2 provides only limited insights with respect to this.

 Other than the above comments, the paper is easy to read and the figures are of good quality.

 I think the authors should address the issues mentioned above to improve their work here, which, as I mentioned above, is interesting and deals with an important issue.

Is the rationale for developing the new method (or application) clearly explained?

Partly

Is the description of the method technically sound?

Partly

Are the conclusions about the method and its performance adequately supported by the findings presented in the article?

Partly

If any results are presented, are all the source data underlying the results available to ensure full reproducibility?

Yes

Are sufficient details provided to allow replication of the method development and its use by others?

No

Reviewer Expertise:

Internet of Things, Smart Cities, Sustainability, Energy Efficiency, Distributed Systems, Pervasive Computing, Gamification, AR/VR

I confirm that I have read this submission and believe that I have an appropriate level of expertise to confirm that it is of an acceptable scientific standard, however I have significant reservations, as outlined above.

## References

[1] : Approaching Sustainability Learning Via Digital Serious Games. *IEEE Transactions on Learning Technologies* .2019;12(3) : 10.1109/TLT.2018.2858770 303-320 10.1109/TLT.2018.2858770

[2] : Making Cool Choices for sustainability: Testing the effectiveness of a game-based approach to promoting pro-environmental behaviors. *Journal of Environmental Psychology* .2017;53: 10.1016/j.jenvp.2017.06.007 20-30 10.1016/j.jenvp.2017.06.007

[3] : Fight the Power! Games, Thermostats, and the Energy Patriarchy. *International Journal of Designs for Learning* .2020;11(2) : 10.14434/ijdl.v11i2.26100 118-129 10.14434/ijdl.v11i2.26100

# Response

**Published:** 2023-08-04

**Authors:** João Cravinho

**Affiliations:** EDP NEW, Portugal

Dear Georgios Mylonas,

 Thank you for your valuable feedback and constructive comments on our manuscript. We appreciate your insights and have made significant improvements to address the concerns raised.

 We agree that the title should better reflect the central aspects of the article. Hence, we have adjusted the title to highlight the use of gamification for improving user engagement with our energy literacy platform. Additionally, we have updated the abstract and introduction to provide a more comprehensive overview of the design and development process, as well as the intended audience for our solution.

 To provide a more comprehensive understanding of the context, we have revised and expanded Sections 2 and 3 to include an in-depth review of existing literature on gamification for energy savings in the residential sector. We have also included relevant comparisons with results from other H2020 projects, highlighting the distinctive features and contributions.

 We acknowledge the need for a more explicit conceptual and strategic approach to the application of gamification. In response, we have included a detailed description of our design approach in the second version of the manuscript. Additionally, we understand the importance of evaluating the effectiveness of our gamification elements. Hence, we have extended Section 3.2.7 to include a summary of our experimental testing sessions and the feedback collected from end-users. These evaluations provide valuable insights into the possible impact and effectiveness of our gamified approach.

 In the context of the use cases in the project, we have included a more complete description in the revised version. We have also provided further clarifications regarding the different user groups, their roles, and how our gamified solution caters to their specific needs and motivations.

 In the revised version, we have also extended Section 3.3 to discuss the limitations of our study and outline potential directions for future research, considering the feedback received. Please bear in mind that all the changes mentioned above relate to the second version of the document, already submitted.

 Once again, we sincerely appreciate your time and effort in reviewing our manuscript. Your input has been instrumental in enhancing the quality and rigor of our work.

 Best regards, João Cravinho

# Reviewer report: Reviewer response for version 1

**DOI:** 10.21956/openreseurope.16396.r32045

**Published:** 2023-07-31

**Authors:** Carlos Vaz De Carvalho

**Affiliations:** 1 Instituto Superior de Engenharia do Porto, Porto, Portugal

The article presents an interesting proposal for the application of gamification in the energy sector. However, the title should better reflect the central aspects of the article (use of gamification to improve the user engagement with a platform meant to develop energy literacy), though. 

 The most relevant problem with the document is that, in all the article we lack a conceptual/strategical approach to the application of gamification. We miss references to gamification frameworks (like Octalysis) and we cannot find the description of an organized and conceptual process that leads from the human motivational triggers or drives (was there any attempt to identify these in the end-users?) towards the selection of the gamification elements. Instead it seems that gamification elements were chosen rather casuistically (probably because, unfortunately, the "norm" for most gamification implementations is the PBL approach where PBL stands for Points, Badges, Leaderboards) and without a real concern for the users' motivations.

 Finally, we also lack an evaluation of the obtained results with the end-users which could demonstrate that these gamification elements were (or not) in fact effective in spite of the aforementioned issues.

Is the rationale for developing the new method (or application) clearly explained?

Yes

Is the description of the method technically sound?

Partly

Are the conclusions about the method and its performance adequately supported by the findings presented in the article?

Partly

If any results are presented, are all the source data underlying the results available to ensure full reproducibility?

No source data required

Are sufficient details provided to allow replication of the method development and its use by others?

Yes

Reviewer Expertise:

Games, Serious Games, Gamification

I confirm that I have read this submission and believe that I have an appropriate level of expertise to confirm that it is of an acceptable scientific standard, however I have significant reservations, as outlined above.

# Response

**Published:** 2023-08-04

**Authors:** João Cravinho

**Affiliations:** EDP NEW, Portugal

Dear Carlos Vaz de Carvalho,

 Thank you for your valuable feedback and constructive comments on our manuscript. We appreciate your insights and have made significant improvements to address the concerns raised.

 We agree that the title should better reflect the central aspects of the article. In the revised version, we have adjusted the title to highlight the use of gamification for improving user engagement with our energy literacy platform. We acknowledge the need for a more explicit conceptual and strategic approach to the application of gamification. In response, we have included a detailed description of our design approach, drawing from the Gamification Model Canvas (GMC) framework and the MDA (Mechanics-Dynamics-Aesthetics) framework. We now discuss the process that leads from the human motivational triggers or drives towards the selection of gamification elements. Additionally, we have considered the taxonomy of player types existing in gamification contexts and the behavioral needs and triggers of our end-users to ensure our gamified design aligns with their motivations. Moreover, to provide a more comprehensive understanding of the context, we have revised and expanded Sections 2 and 3 to include an in-depth review of existing literature on gamification for energy savings in the residential sector. We understand the importance of evaluating the effectiveness of our gamification elements.

 We have extended Section 3.2.7 to include a summary of our experimental testing sessions and the feedback collected from end-users. These evaluations provide valuable insights into the possible impact and effectiveness of our gamified approach.

 In the revised version, we have extended Section 3.3 to discuss the limitations of our study and outline potential directions for future research, considering the feedback received.

 Once again, we sincerely appreciate your time and effort in reviewing our manuscript. Your input has been instrumental in enhancing the quality and rigor of our work. Best regards, João Cravinho

# Reviewer report: Reviewer response for version 1

**DOI:** 10.21956/openreseurope.16396.r31814

**Published:** 2023-07-14

**Authors:** Inma Rodriguez

**Affiliations:** 1 Department of Mathematics and Computer Science, UBICS Research Institute, Barcelona, Spain

This paper presents an interesting contribution in the context of gamified solutions for energy awareness and literacy. It introduces a gamification module designed to promote and foster sustainable behavior and user learning on the Smart2B platform. This platform is a cloud-based energy management application directed to different stakeholders (buildings’ occupants, administrators, groups of citizens,..). The paper is well written and structured. Nevertheless, I think it is necessary an experimental evaluation of the system to test the effectiveness of the gamified design.

 Another remark is that the paper could expand the related work section. The current version of the paper does not give sufficient information about the existing literature on gamification for energy awareness. It would be valuable to discuss the similarities and differences between this contribution and others in the field.

 Figure 1 presents the conceptualization of the gamification with a puzzle-like diagram. However, the authors do not mention any specific design framework they followed to design their gamified system. The literature reports several game design and gamification design frameworks such as MDA [1], GMC [2]. It would be helpful if authors explain how they arrived at the design and whether they drew inspiration from any of these frameworks. Additionally, there is a distinction between shallow (extrinsic rewards, PBL-Points Badges Levels) and deep (further than PBL) gamification [3]. Authors could rationale what type of gamification they are contributing with.

 [1] Hunicke, R., LeBlanc, M., & Zubek, R. (2004, July). MDA: A formal approach to game design and game research. In *Proceedings of the AAAI Workshop on Challenges in Game AI* (Vol. 4, No. 1, p. 1722).

 [2] Escribano, F., & Cp, A. (2010). Gamification model canvas evolution for design improvement: Player profiling and decision support models. *Fundación Iberoamericana Del Conocimiento*, 1-6.

 [3]Lieberoth, A. (2015). Shallow gamification: Testing psychological effects of framing an activity as a game. *Games and Culture*, *10*(3), 229-248.

 Regarding the user profile of smart2B users, it is important to define this profile and then present gamification elements that fit it. Moreover, authors does not refer to player types taxonomies [4] and how they can assist in considering mechanics for users that like to play differently. This way the design would consider the most effective game design elements for targeted users.

 [4] Tondello, G. F., Wehbe, R. R., Diamond, L., Busch, M., Marczewski, A., & Nacke, L. E. (2016, October). The gamification user types hexad scale. In *Proceedings of the 2016 annual symposium on computer-human interaction in play* (pp. 229-243).

 When the authors mention a UI tailored to users' needs, it would be beneficial to provide an exact explanation of what they mean. Does it imply an adaptive UI in some way? Or do they mean that different user profiles have different UI elements to interact with?

 The paper mentions the "game narrative" several times, but its definition is not clear. Typically, a game narrative is associated with a scenario, characters, plot, etc., which is not explicitly considered in this paper. Some clarification is needed to inform the reader about how the concept is being used by the authors.

 To enhance the paper, it would be great to find the definition of terms that the reader is not sure about their meaning, for example, Smart Readiness Indicator (SRI) level (do they refer to smartmeters?) and energy flows (do they refer to input and output power when houses have installed solar energy?).

 As minor remarks:

- In the context of software design, concretely in UML (Unified modeling language), the” use case diagram” is well known, but it does not correspond exactly to what Figure 2 depicts. I would recommend updating the caption.
- Similarly, relating to caption of Figure 1, I think that the Figure 1 does not describe the conceptualization of game-design elements (which is part of the gamification design) but the conceptualization of the gamification.
- When the authors mention "each of the three dimensions that the occupants can adjust," the dimensions are enumerated later in the text, but it would be more effective to introduce these dimensions in parentheses or a similar manner right after the initial phrase.

Is the rationale for developing the new method (or application) clearly explained?

Yes

Is the description of the method technically sound?

Yes

Are the conclusions about the method and its performance adequately supported by the findings presented in the article?

Partly

If any results are presented, are all the source data underlying the results available to ensure full reproducibility?

No

Are sufficient details provided to allow replication of the method development and its use by others?

Partly

Reviewer Expertise:

HCI design and methods, Gamification and game-based learning

I confirm that I have read this submission and believe that I have an appropriate level of expertise to confirm that it is of an acceptable scientific standard, however I have significant reservations, as outlined above.

## References

[1] : MDA: A Formal Approach to Game Design and Game Research. *Proceedings of the AAAI Workshop on Challenges in Game AI* .2004;4(1) : Reference source

[2] : Gamification Model Canvas Evolution for Design Improvement: Player Profiling and Decision Support Models. *GECON.es – Fundación Iberoamericana del Conocimiento* .2010; Reference source

[3] : Shallow Gamification. *Games and Culture* .2015;10(3) : 10.1177/1555412014559978 229-248 10.1177/1555412014559978

[4] : The Gamification User Types Hexad Scale. *CHI PLAY '16: Proceedings of the 2016 Annual Symposium on Computer-Human Interaction in Play* .2016;229-243 Reference source

# Response

**Published:** 2023-08-04

**Authors:** João Cravinho

**Affiliations:** EDP NEW, Portugal

Dear Inma Rodriguez,

 First and foremost, we would like to express our gratitude for the comprehensive review report. Your insightful comments and valuable feedback have proven crucial for the improvement and the quality of our manuscript.

 Regarding the experimental evaluation of the gamified module, we have taken your suggestion seriously and have now extended Section 3.2.7 to include a summary of our experimental testing sessions, along with the feedback we have collected so far. This additional information should provide more insights into the effectiveness of our gamified design. Furthermore, we acknowledge the need for a more thorough discussion in the related work section.

 We have revised and expanded Sections 2 and 3 to include a more comprehensive review of existing literature on gamification for energy savings in the same context. Additionally, we have now explained how our design approach drew inspiration from the Gamification Model Canvas (GMC) framework and the MDA (Mechanics-Dynamics-Aesthetics) framework. We have also included the rationale behind the type of gamification we are contributing with, drawing from the distinction between shallow and deep gamification. Moreover, the taxonomy of player types has been mentioned as well as the behavioral needs and triggers of users, key factors for the design of this module.

 Regarding the UI tailored to users' needs, we have clarified our intentions, explaining that it implies an adaptive UI in some instances, while in other cases, it refers to different user profiles having distinct UI elements to interact with. In the same note, we have taken the feedback about the correct terminology (e.g., game narrative) and have made the changes accordingly. Additionally, we have added definitions for terms that might be unclear to readers, such as the Smart Readiness Indicator (SRI) level and energy flows.

 Regarding the minor remarks, we have updated the caption for the "use case diagram" to reflect the context more accurately. Similarly, we have revised the caption and content of Figure 1. Furthermore, we have made the dimensions that occupants can adjust more explicit by introducing them immediately after the initial phrase.

 Lastly, we have extended section 3.3 to include a discussion of the present study's limitations and potential areas for future work.

 Once again, we express our appreciation for your thorough review, and we hope that these revisions address the concerns raised effectively.

 We are confident that the enhanced manuscript now provides a more comprehensive and well-explained presentation of our work. Thank you for your time and consideration. Sincerely, João Cravinho
